# Leveraging Chelating Amido Ligands to Support Metal–Metal Bonding in Dinuclear Cr(II) Complexes

**DOI:** 10.1021/acs.inorgchem.6c00424

**Published:** 2026-03-05

**Authors:** Janadhi L. Ananda Nakath Durage, Joan Cardona, Soumen Sinhababu, Darby H. Duffy, Daniel Martinez, Matthew P. Shores, Daniel K. Unruh, Bess Vlaisavljevich, Scott R. Daly

**Affiliations:** † Department of Chemistry, 4083 The University of Iowa, Iowa City, Iowa 52442, United States; ‡ Departament de Química Inorgànica i Orgànica and Institut de Recerca de Química Teòrica i Computacional, Universitat de Barcelona, Diagonal 645, Barcelona 08028, Spain; § Department of Chemistry, 3447 Colorado State University, Fort Collins, Colorado 80523, United States; ∥ The University of Iowa, Office of the Vice President for Research, 2660 UCC, Iowa City, Iowa 52242, United States

## Abstract

Herein, we report a series of Cr­(II) complexes containing triaryl, tetradentate ligands derived from *o*-phenylenediamide that illuminate how rigid, chelating amido ligands facilitate Cr–Cr bonding. The aminolysis reaction of Cr­[N­(SiMe_3_)_2_]_2_(thf)_2_ with H_2_(**L1**)—a protonated N_4_ proligand containing flanking NMe_2_ groups—yielded square planar and mononuclear Cr­(**L1**) (**1**), whereas the same reaction with N_2_S_2_ and N_2_O_2_ proligands with flanking SMe and OMe groups yielded dinuclear [Cr­(**L2**)]_2_ (**2**) and [Cr­(**L3**)]_2_ (**3**). The structures of **2** and **3** revealed Cr–Cr distances of 2.3356(6) and 2.3481(5) Å, consistent with metal–metal bonding, which was confirmed by the complete active space methods. The theoretical results suggest that Cr–Cr bonding is assisted by the chelating nature of the bridging amido ligands, which fold the dinuclear structure and orient the metals so that side-on overlap of Cr 3d orbitals can occur. Variable-temperature SQUID magnetometry and spectroscopic data (e.g., UV–vis-NIR, Raman, and IR) reported for **1–3** show differences indicative of the change in nuclearity and electronic structure. Collectively, these results reveal bridging amido ligand characteristics that support metal–metal bonding with Cr­(II), and they help account for the wide range of metal–metal distances observed in dinuclear (or binuclear) Cr­(II) complexes containing Cr_2_N_2_ cores.

## Graphical abstract

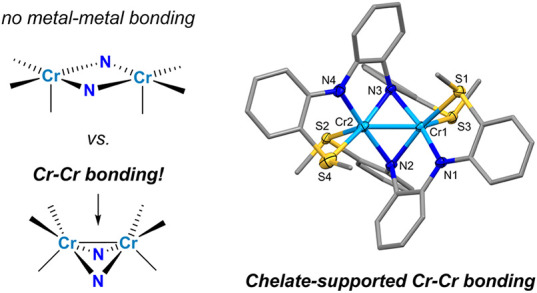

## Introduction

Chromium is featured prominently in efforts aimed at forming metal–metal bonds with first-row transition metals.^1–5^ It has long been known to form dinuclear complexes containing unsupported metal–metal multiple bonds,^4^ with bond orders as high as five being achieved.^6–9^ Dichromium complexes supported by bridging ligands are also known, but these ligands tend to reduce bond orders and can make it difficult to assess the presence of metal–metal bonds. Bond orders are often inferred initially based on Cr–Cr distances and related analyses such as the formal shortness ratio (fsr),^5^ but these can be unreliable for metal–metal bond assessments. In this context, it is well recognized that bridging ligands play a critical role in modulating dinuclear structure, metal–metal bonding, and magnetic properties, but disentangling the interplay between these effects remains a significant challenge.^10^

Dichromium complexes containing bridging amido ligands, especially those containing diamond Cr_2_N_2_ cores, highlight how bridging ligands can yield dramatic differences in metal–metal distances. As shown with representative examples in Figure 1a, Cr–Cr distances in amido-bridged complexes are typically ≥2.8 Å,^11–21^ but there are some examples where they fall well below this threshold.

**Figure 1:**
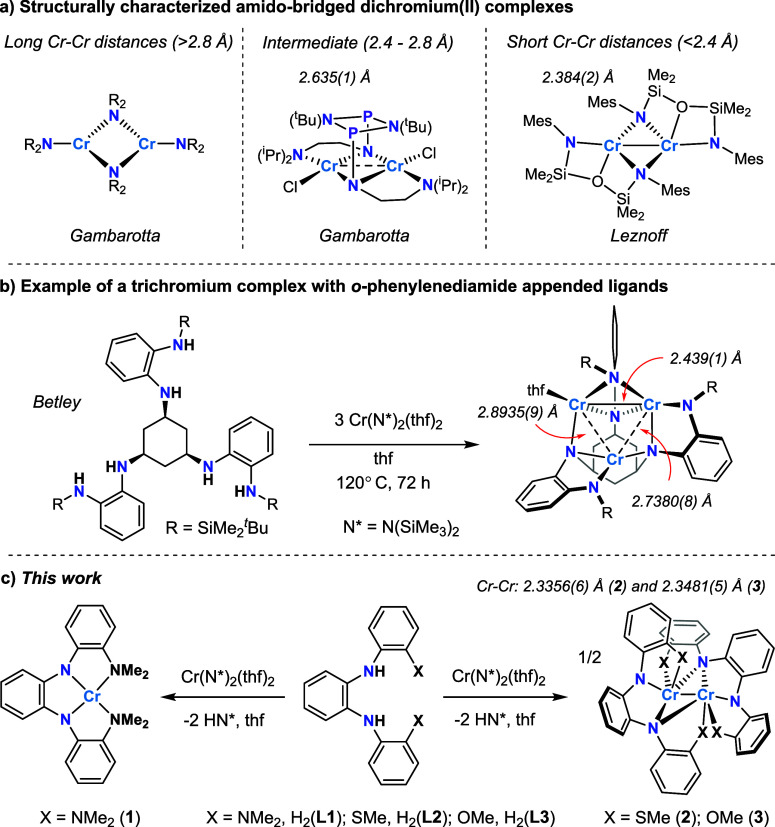
a) Representative examples of structurally characterized dichromium­(II) complexes containing bridging amido groups and a Cr_2_N_2_ diamond core. b) Example of a trichromium complex containing a Cr_2_N_2_ diamond core. c) Synthesis and structures of Cr complexes **1–3**.

For example, Gambarotta and coworkers reported Cr–Cr distances of 2.635(1) and 2.709(1) Å for the two molecules of {CrCl­[(μ-N)­(^t^Bu)]_2_[PNCH_2_CH_2_N­(^i^Pr)_2_]_2_}_2_ in the asymmetric unit cell (Figure 1a).^22^ Leznoff and coworkers reported an even shorter Cr–Cr distance of 2.384(2) Å in the structure of [Cr­[MesN­(SiMe_2_)]_2_O]_2_ (Mes = 2,4,6-trimethylphenyl) with a tridentate (NON)^2–^ ligand.^23^ This Cr–Cr distance is remarkably short, and the bridging amido ligands yielded significant antiferromagnetic coupling between the metals, as indicated by an effective magnetic moment (μ_eff_) of 2.38 μ_B_.^23^ Apart from dinuclear complexes, other notable examples of short Cr–Cr distances supported by bridging amido ligands have been found in trichromium clusters reported by Betley and coworkers (Figure 1b).^24^ For example, the complex (^tbs^L)­Cr_3_(thf) is supported by an *o*-phenylenediamide-based hexadentate ligand ([^tbs^L]^6–^ = [1,3,5-C_6_H_9_(NC_6_H_4_-*o*-NSiMe_2_^t^Bu)_3_]^6–^), and this cluster has one short Cr–Cr distance of 2.439(1) Å, and two longer Cr–Cr distances of 2.7380(8) and 2.8935(9) Å.^24^ Abstracting thf from (^tbs^L)­Cr_3_(thf) to form (^tbs^L)­Cr_3_ caused the shortest Cr–Cr distance to decrease further to 2.3638(6) Å.

Computational methods are often used to assess and rationalize structural differences in complexes containing metal–metal bonds, but this continues to be a challenge for first-row transition metals, especially when using conventional methods such as density functional theory (DFT) that can fail to sufficiently recover electron correlation. These challenges have been well-documented in complexes with unsupported metal–metal bonds,^25,26^ but they are even more daunting when bridging ligands are present, like those in complexes containing Cr_2_N_2_ cores. These challenges take two forms. In some systems, the metal–metal bonding with first-row transition metals, particularly with Cr, is multiconfigurational, leading to significant population of the antibonding orbitals.^25,27^ As a result, the Cr–Cr bond strength in many of these complexes is relatively weak, even for those containing formally quadruple Cr–Cr bonds. For example, Gambarotta and coworkers showed that simply adding pyridine (py) to Cr_2_(tmtaa)_2_ (tmtaa = dibenzotetramethyltetraaza[14]­annulene dianion) cleaves the Cr–Cr quadruple bond to form mononuclear Cr­(tmtaa)­(py)_2_.^28^ In other systems, the potential energy surface between different structures can be very flat, requiring careful treatment of dispersion effects beyond the ability of even dispersion-corrected DFT approaches. The systems with Cr_2_N_2_ cores are expected to be intermediate, requiring both a multiconfigurational method and careful accounting for dispersion effects.

Herein, we address the fundamental question of what accounts for the differing Cr–Cr distances observed in dinuclear Cr_2_N_2_ complexes. We report a series of Cr­(II) complexes containing triaryl tetradentate ligands synthesized previously with Ru and Fe (**L1** and **L2** with X = NMe_2_ and SMe),^29,30^ as well as a new ligand with flanking OMe donor groups (**L3**). Building on our analysis of diiron complexes reported recently,^29^ we show how Cr complexes of different nuclearity are obtained with **L1** (mononuclear) and **L2** and **L3** (dinuclear). The dinuclear complexes have remarkably short Cr–Cr distances consistent with metal–metal bonding, and we demonstrate how second-order multireference methods (CASSCF/CASPT2) can accurately describe the nature of the Cr–Cr bonds. The results reveal how structural constraints of chelating amido ligands govern the structure and availability of metal–metal bonding in Cr_2_N_2_ complexes.

## Results

The protonated N_4_ and N_2_S_2_ proligands, H_2_(**L1**) and H_2_(**L2**), were synthesized as described previously by Buchwald–Hartwig cross-coupling of *o*-phenylenediamine with two equivalents of 2-bromo-*N,N*-dimethylaniline or 2-bromothioanisole, respectively.^2,3^ The N_2_O_2_ proligand, H_2_(**L3**), was prepared similarly from 2-bromoanisole and is described here for the first time. After extraction, H_2_(**L3**) was purified by silica gel column chromatography and isolated in 60% yield. The ^1^H and ^13^C NMR spectra in CDCl_3_ revealed singlets assigned to the OMe resonances at δ_H_ = 3.84 and δ_C_ = 55.63 ppm, respectively. The N–H groups yielded a broad resonance at δ_H_ = 6.08 ppm in the ^1^H NMR spectrum. The remaining NMR peak positions and integrations support the chelating triaryl framework and proposed formulation. Single-crystal X-ray diffraction (XRD) data collected on H_2_(**L3**) further corroborated its structure and connectivity (see the SI for full details).

Stirring solutions of H_2_(**L1**) and H_2_(**L2**) with Cr­[N­(SiMe_3_)_2_]_2_(thf)_2_ in thf and subsequent workup and crystallization by vapor diffusion yielded purple crystals of **1** and dark-brown crystals of **2** that were suitable for XRD studies. Reactions of Cr­[N­(SiMe_3_)_2_]_2_(thf)_2_ with H_2_(**L3**) and similar workup yielded dark-green crystals of **3** in low yield as well as an assortment of other difficult-to-separate products, as we will discuss in more detail below.

The single-crystal XRD data for **1** with X = NMe_2_ revealed a mononuclear Cr­(II) complex with a square-planar coordination geometry (τ_4_ = 0;^31^ Figure 2). The unique Cr–N1 and Cr–N2 bond distances of 1.970(1) and 2.163(1) Å are consistent with X-type amide and L-type amine donors. The N1–Cr–N1 and N1–Cr–N2 bond angles are 81.47(7)° and 80.26(4)°, respectively, revealing the distortion from 90° in perfect square planarity. The square-planar geometry in **1** is similar to those observed for a series of Cr­(II) diketiminate (nacnac) complexes.^32^ For comparison, these Cr­(nacnac)_2_ complexes had Cr–N distances that ranged from 2.050(1) to 2.094(1) Å.

**Figure 2:**
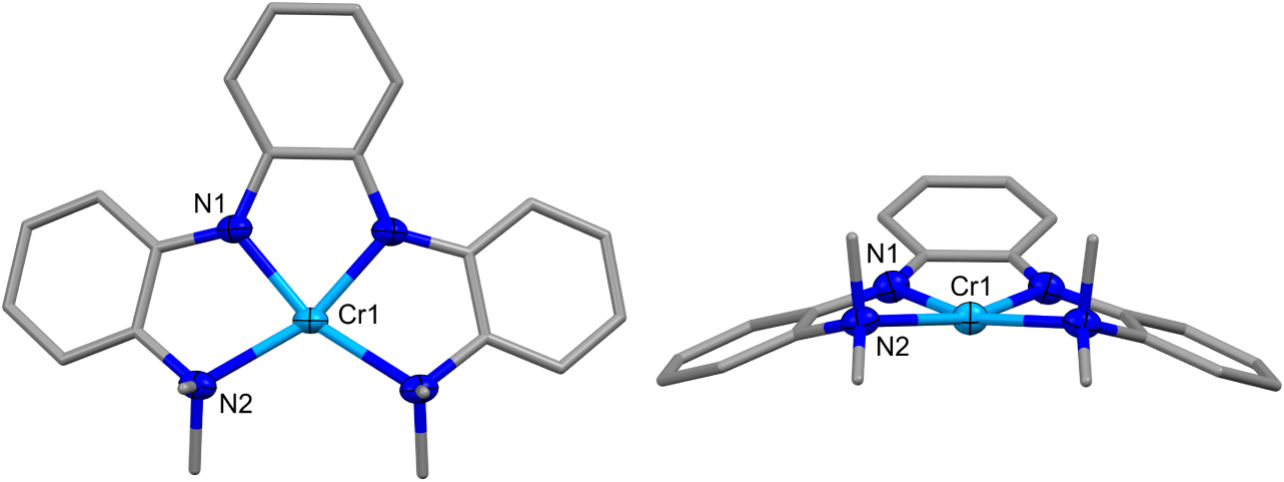
Molecular structure of **1**. Ellipsoids for noncarbon atoms are drawn at 50% probability. Carbon atoms are drawn as capped sticks. Hydrogen atoms have been omitted. Select distances and angles: Cr1–N1 = 1.970(1) Å; Cr1–N2 = 2.163(1) Å; N1–Cr1–N1 = 81.47(7)°; N1–Cr1–N2 = 80.26(5)°; N2–Cr1–N2 = 116.05(6)°.

In contrast to those of **1**, single-crystal XRD data collected for **2** and **3** revealed dinuclear complexes with a puckered Cr_2_N_2_ core (Figures 3 and 4). The Cr–Cr distances in **2** and **3** are nearly identical at 2.3356(6) and 2.3481(5) Å, and both are shorter than those reported for other dinuclear Cr complexes containing bridging amido ligands like those shown in Figure 1. The Cr–Cr distances in **2** and **3** yield a formal shortness ratio of 1.00, suggesting the presence of a Cr–Cr bond. This was corroborated by the theoretical calculations described below. The bridging Cr–N distances are relatively symmetric, ranging from 2.062(2)–2.091(2) Å for **2** and 2.059(1)–2.118(1) Å for **3**. This is quite different from the same Fe–N distances reported previously with dinuclear [Fe_2_(**L**_**2**_)_2_],^29^ which showed more asymmetry in the diamond core; the Fe–N distances ranged from 2.051(2) to 2.286(2) Å. The Cr–N–Cr angles are rather acute at 68.28(6)° and 68.91(6)° for **2** and 68.01(4)° and 69.12(5)° for **3**.

**Figure 3:**
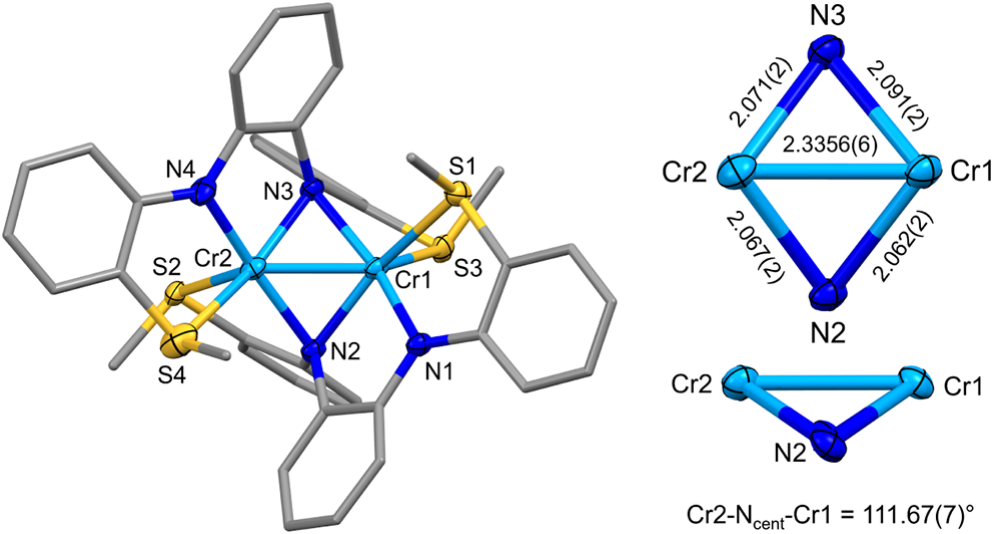
Molecular structure of **2** looking down the *C_2_* axis. Ellipsoids for noncarbon atoms are drawn at 50% probability. Carbon atoms are drawn as capped sticks. Hydrogen atoms and disordered components have been omitted. Distances are reported in Å.

**Figure 4:**
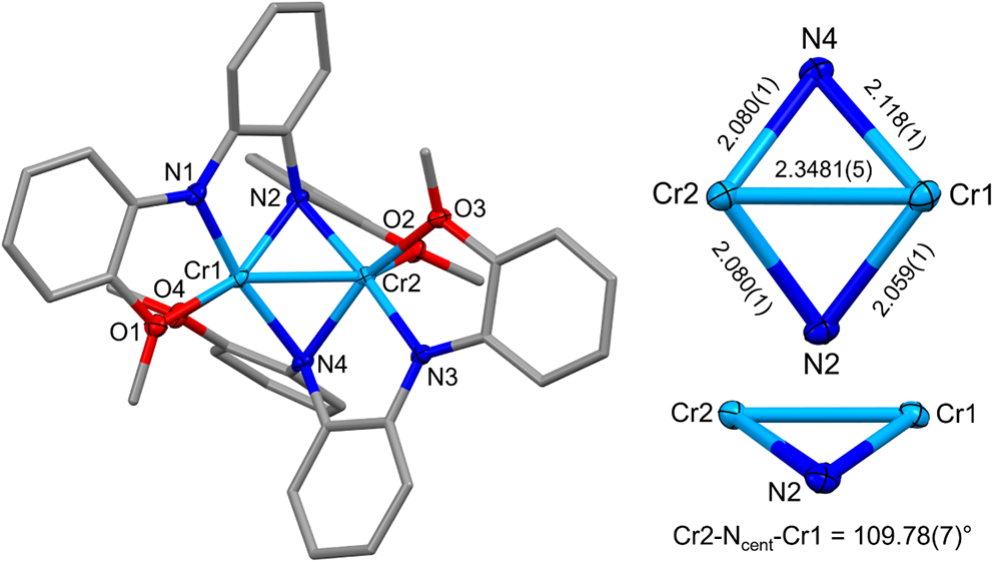
Molecular structure of **3** looking down the *C_2_* axis. Ellipsoids for noncarbon atoms are drawn at 50% probability. Carbon atoms are drawn as capped sticks. Hydrogen atoms and disordered components have been omitted. Distances are reported in Å.

Complexes **2** and **3** are chiral and possess axial *C*_2_ symmetry. Both complexes crystallize in the centrosymmetric space group *P*2_1_/*c*, so both enantiomers are present in the crystals. Ignoring the Cr–Cr bond, the coordination geometry around each Cr ion is square pyramidal. Calculation of the τ_5_ values^33^ for each metal in **2** (τ_5_ = 0.00/0.08) and **3** (τ_5_ = 0.16/0.26) reveals that **3** has a higher degree of distortion away from square pyramidal, which may account for its increased reactivity (see below). The two bridging N atoms form a hinge that connects the square pyramids (Chart [Fig chart1]). The deviation of the hinge from 180° has been referred to as the hinge distortion,^34^ and it has been reported previously in so-called roof-shaped dicopper complexes (roof-shaped indicating the fold in the hinge).^34–37^ The hinge angle is quantified and defined here as the Cr–N_cent_–Cr angle, where N_cent_ is the centroid position between the two bridging N atoms that form the hinge. The Cr–N_cent_–Cr angles are 111.67(7)° (**2**) and 109.78(7)° (**3**) (Chart [Fig chart1]). For comparison, the Cr–N_cent_–Cr angle for [Cr­[MesN­(SiMe_2_)]_2_O]_2_ shown in Figure 1a is 109.1(2)°. Like the bridging Cr–N–Cr angles, these Cr–N_cent_-Cr angles are far more acute than those typically observed in roof-shaped dicopper complexes (133°–161°).^34–37^

**1 chart1:**
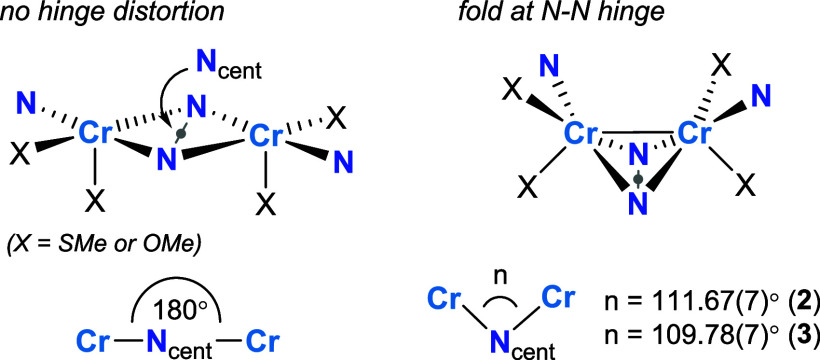
Comparison of **2** and **3** with and without a Fold at the N–N Hinge

The reproducible isolation of **3** with satisfactory purity for follow-up spectroscopic and magnetism studies proved to be a significant challenge. We quickly discovered that **3** is readily oxidized by chlorinated solvent vapor (likely CH_2_Cl_2_) present in our glovebox atmosphere. A secondary species was isolated and identified as a tetrameric complex featuring two chloride ligands bridging two dimer units (**4**; Figure 5).

**Figure 5:**
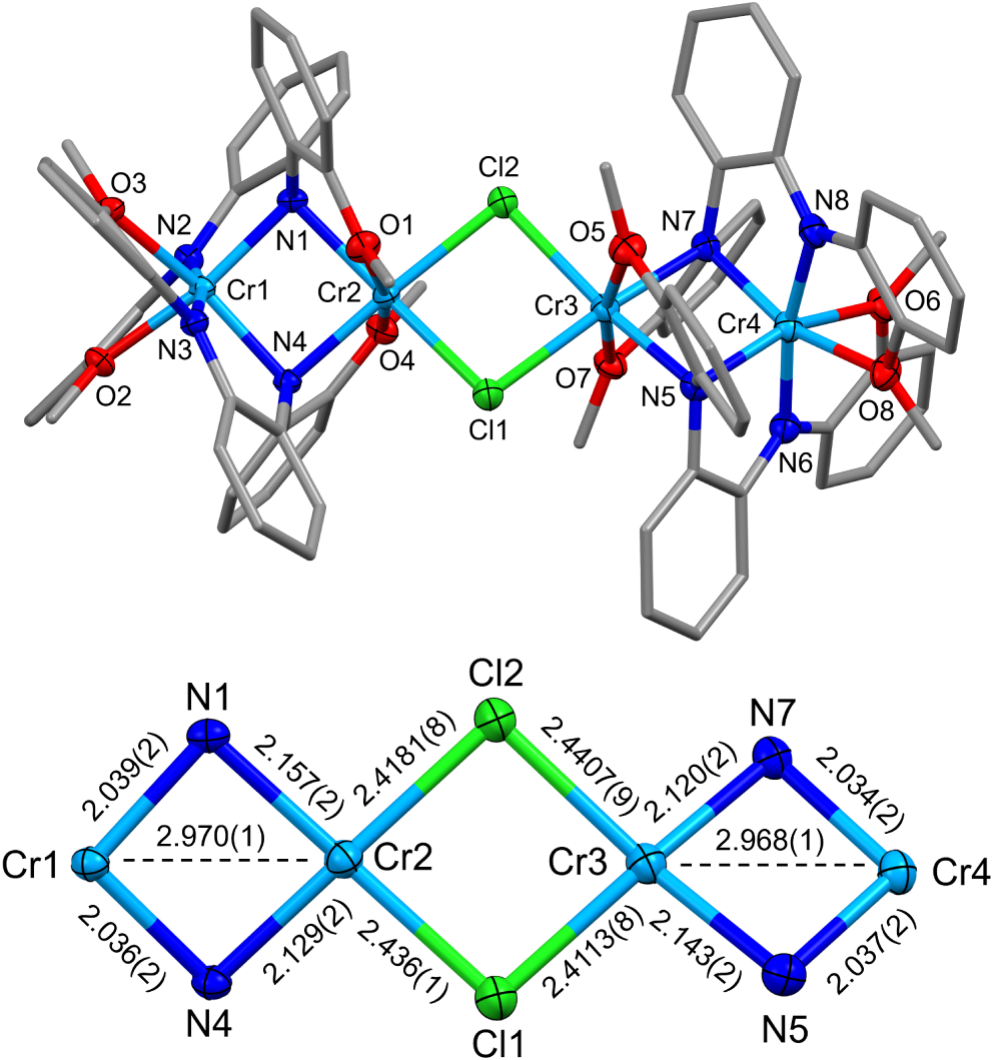
Molecular structure of **4**. Ellipsoids for noncarbon atoms are drawn at 50% probability. Carbon atoms are drawn as capped sticks. Hydrogen atoms have been omitted. Distances shown are reported in Å.

The Cr–Cr distance increased from 2.3481(5) Å in **3** to an average distance of 2.969(1) Å in **4**, and the changes in Cr–N and Cr–O bond distances are consistent with the oxidation of one of the divalent Cr ions in the Cr_2_N_2_ core. Based on structural metrics, **4** appears to be a Robin–Day class I complex^38^ with formal Cr^II^Cr^III^(μ-Cl)_2_Cr^III^Cr^II^ oxidation state assignments. The Cr^III^–Cl distances range from 2.4113(8) to 2.4407(9) Å and show slight asymmetry with a long and short distance associated with each bridging chloride. For comparison, these distances are only slightly longer than those reported for Cr^II^ complexes like [Cr­[N­(SiMe_3_)_2_]­(thf)]_2_(μ-Cl)_2_,^139^ which has bridging chloride distances of 2.4001(6) and 2.4234(5) Å. Additionally, this complex can undergo hydrolysis in the presence of adventitious water to replace one of the bridging chlorides with a hydroxyl ligand (Figure S2; SI).

It is not immediately clear why **3** undergoes reactions with chlorinated solvents, whereas dinuclear **2** does not, though similar chlorination reactions have been reported with other dinuclear Cr–Cr bonded complexes and CH_2_Cl_2_.^39^ One possibility is that the longer C–S bonds in **L2** can envelop more of the Cr coordination sphere and better protect the dinuclear Cr_2_N_2_ core compared to the shorter C–O bonds in **L3**. This is consistent with the greater τ_5_ distortion from the square pyramidal structure described above for the Cr ions in **3**. We also note changes in the orientation of the methyl groups in the structures of **2** and **3** (Figures 3 and 4), which may also alter the degree of steric protection at the metals. To explore this hypothesis further, we quantified the percentage buried volume (%V_bur_) for complexes **2** and **3** using ChimeraX.^40,41^ Complex **2** with SMe flanking donor groups exhibited a %V_bur_ of 92.7%, while **3** with OMe donor groups showed a lower value of 85.0% (Figure 6). The decreased steric protection in **3** may account for the differences in reactivity, but we cannot rule out ligand field and related electronic effects. Another possibility is that the speciation and nuclearities of **2** and **3** are different in solution. However, we note that **1** does not show any evidence of reactivity with CH_2_Cl_2_, despite being monomeric and Cr being readily accessible. This may indicate that both metals are required for the reaction to occur.

**Figure 6:**
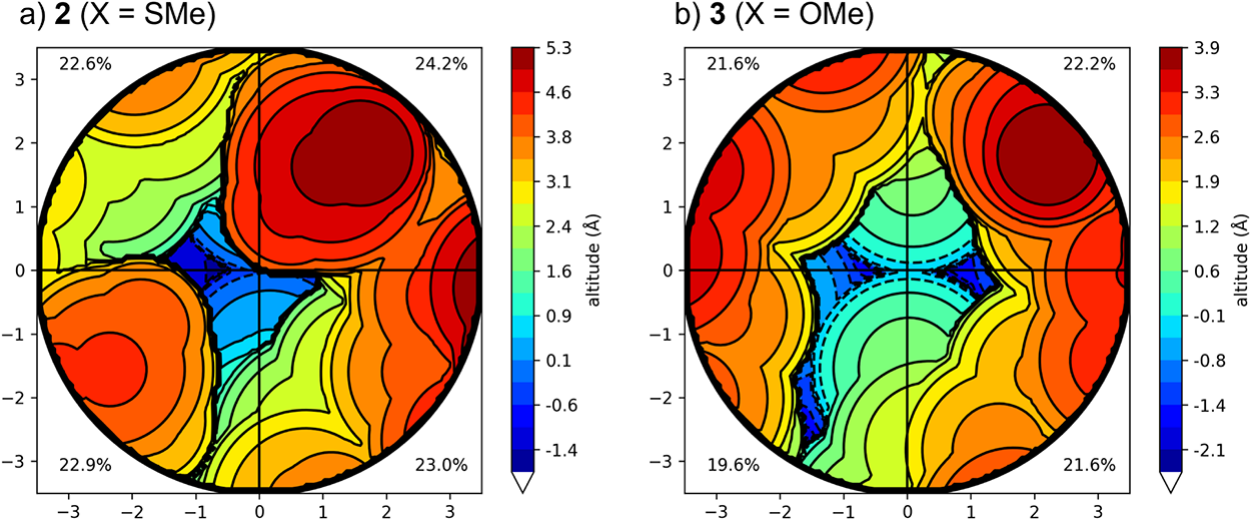
Calculated steric maps around the dinuclear Cr–Cr core of **2** (a; left) and **3** (b; right) from buried volume calculations (%V_bur_). The origin (0,0) indicates the center of the Cr–Cr bond. The differing colors indicate altitude in Å from the origin.

Attempts to prepare **3** with rigorous absence of chlorinated solvents resulted in material that contained **3** but also other side products that were difficult to identify due to their paramagnetism and lack of crystallinity. Despite repeated attempts, the material that was isolated never produced satisfactory elemental analysis data consistent with only **3**. Given the difficulty in preparing samples of **3** with sufficient purity for spectroscopic and magnetic studies, the following section will focus exclusively on a comparative analysis of **1** and **2**.

### Spectroscopic Analysis

Solution UV–vis-NIR spectroscopy data were collected for **1** and **2** in thf to investigate electronic structure variations due to the presence and absence of the dinuclear Cr_2_N_2_ core. As in the solid state, differences in the two solutions were visually apparent based on their color (purple for **1** and dark brown for **2**). The spectra of both complexes were dominated by intense charge transfer bands in the UV region. The spectrum of **1** exhibits transitions at 275, 303, 345, and 375 nm. These were shifted to lower energy and were more intense for **2** at 296 and 351 nm (Table 1; Figure S14, SI). An additional and relatively intense feature was also observed for dinuclear **2** at 455 nm (ε = 4220 M^–1^ cm^–1^) that was not observed in the spectrum of **1** (Figure 7). This absorption may be associated with the Cr–Cr bond; it is strikingly similar to a diagnostic metal–metal bonding feature reported at 450 nm for a singly bonded dichromium complex.^42^ As in our comparison of **1** and **2**, this feature disappeared in the corresponding mononuclear system. Lower energy transitions in the visible region were notably weaker in intensity in both spectra, as expected for d–d transitions. Some of these absorptions were remarkably similar, apart from a broad absorption centered close to 523 nm in the spectrum of **1** and a weak NIR absorption at 1146 nm (ε = 55 M^–1^ cm^–1^) consistent with the mononuclear structure (Figure 7 inset). A similar NIR absorption was reported previously for square planar Cr­(II) and was assigned as a d → d transition.^43^

**Figure 7:**
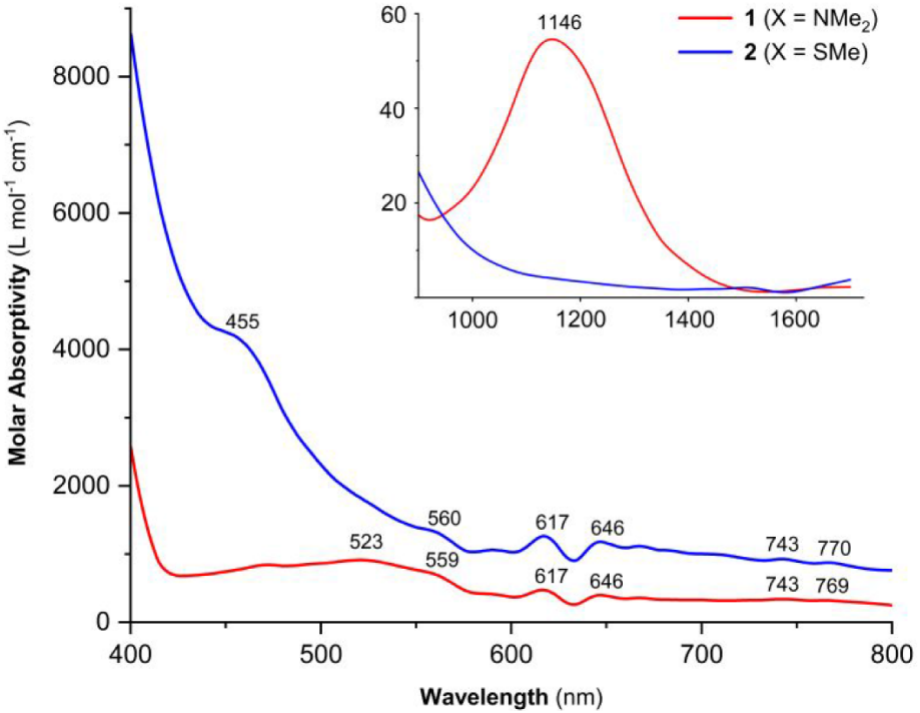
Visible spectra of **1** (X = NMe_2_) and **2** (X = SMe) in thf at room temperature. Inset: NIR spectra. Full UV–vis spectra are provided in the SI.

**Table 1:** UV–Vis Data for **1** and **2** Collected in thf

Cr(**L1**), **1**		[Cr_2_(**L2**)_2_], **2**	
λ_max_ (nm)	ε (M^–1^ cm^–1^)	λ_max_ (nm)	ε (M^–1^ cm^–1^)
275	10,300	296	29,000
303	10,500	351	26,000
345	11,500	455	4,220
375	9390	560	1,340
523	890	617	1,270
559	730	646	1,200
617	470	743	930
646	400	770	860
743	340		
769	290		
1146	55		

The spectral similarities with some of the transitions observed in the solution UV–vis spectra for **1** and **2** raised the possibility that these complexes form other species in thf, perhaps with similar structures. One could envision that thf breaks up dinuclear **2** to some extent in solution to form a mononuclear species. If it occurs, these putative species could be in equilibrium with **1** and **2**, the species that ultimately crystallize from thf solutions. To assess this possibility, we collected the solid-state diffuse-reflectance UV–vis spectra of **1** and **2**. As observed in solution, **1** and **2** display similar spectra in the solid state, though the transitions are very broad, especially for **2** (Figure S16; SI), making it difficult to make any conclusive assessments.

Solid-state IR and Raman data were collected on **1** and **2** to assess the differences in structure and to evaluate the presence of Cr–Cr bonding in **2**. The IR spectra showed similar features in the fingerprint region from 600 to 1700 cm^–1^ (Figure S12; SI), whereas the Raman data revealed more pronounced differences (Figure 8). Perhaps the most notable are clusters of vibrations observed for **2** between 250 and 450 cm^–1^ that are absent in the spectrum of **1**. For comparison, most Raman-active Cr–Cr stretching frequencies reported previously have been those for dichromium complexes with short Cr–Cr quadruple or quintuple bonds.^44–48^ Reported Raman ν_(Cr–Cr)_ bands for these metal–metal multiple-bonded species generally fall within the ∼300–600 cm^–1^ region.^44–48^

**Figure 8:**
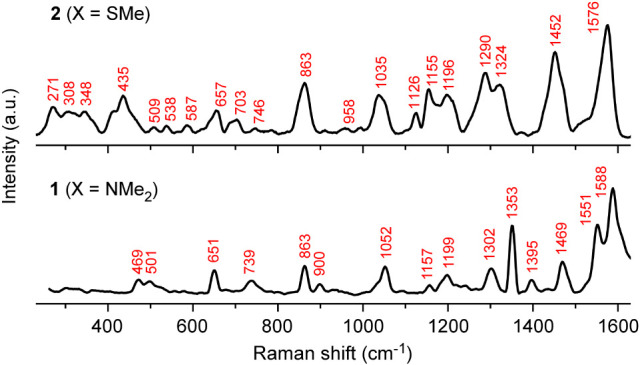
Raman spectra of **1** (X = NMe_2_) and **2** (X = SMe).

The lower-energy Raman vibrations observed for **2** between 250 and 450 cm^–1^ are consistent with those expected for weaker Cr–Cr bonds, but some of these absorptions can likely be assigned to bending modes involving the SMe groups on the **L2** ligand. The Raman spectrum of H_2_(**L2**) revealed several absorptions at 264, 284, 305, and 398 cm^–1^ that are not observed for H_2_(**L1**) or H_2_(**L3**) (Figure S13; SI). Similar Raman features have also been reported previously for thioanisole at 327 and 415 cm^–1^.^49^ Even if these overlapping features were not present, definitive assignments of Cr–Cr vibrational modes are often difficult for complexes like **2** because these metal–metal stretches can couple with the bridging N atoms.^46^ These assignments are also notoriously difficult to verify computationally because of the challenges of accurately modeling weak metal–metal bonding using DFT, as described in the calculations section below.

### Magnetic Studies

Magnetic susceptibility data were collected on **1** and **2** at 0.1 T using a SQUID magnetometer to assess the influences of nuclearity and Cr–Cr interactions on the magnetic properties. Data collected for mononuclear **1** reveal an *S* = 2 species consistent with a high-spin d^4^ configuration (Figure 9). The variable temperature study reveals that **1** behaves as a typical paramagnet. The data were modeled with *g* = 1.91 and small amounts of intermolecular antiferromagnetic exchange coupling (*zJ* = −0.4 cm^–1^) and significant temperature independent paramagnetism (TIP = 580 × 10^–6^ cm^3^·mol^–1^), the latter of which is consistent with a high-spin system where electronic configuration may be fluid owing to small differences in orbital energies.

**Figure 9:**
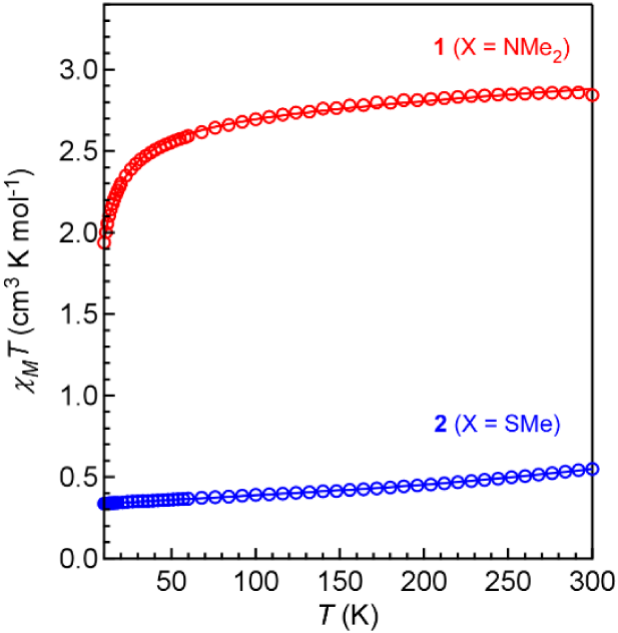
Variable-temperature (zero-field-cooled (zfc)) dc magnetic susceptibility data for restrained polycrystalline samples of **1** (red) and **2** (blue) collected under a 0.1 T applied dc field. The solid lines represent fits to the data. Parameters from best fits: **1**: *S* = 2, *g* = 1.914(1), intermolecular coupling (*zJ*) = −0.4 cm^–1^, temperature-independent paramagnetism (TIP) of 580 × 10^–6^ cm^3^/mol. **2**: *S*_1_ = *S*_2_ = 2, *g*_1_ = *g*_2_ = 1.91(1), *J* = −410(3) cm^–1^, TIP = 650 × 10^–6^ cm^3^/mol; monomeric Cr­(II) *S* = 2 “impurity” set at 11%.

The variable temperature data collected for dinuclear **2** are dramatically different compared to those for **1** (Figure 9). Dinuclear **2** exhibits significant intramolecular antiferromagnetic exchange coupling that was modeled with a *J* value (using the −2*J* formalism) of −410(3) cm^–1^. For comparison, the dinuclear Fe­(II) complex [Fe_2_(**L2**)_2_] reported previously with the same ligand showed significantly weaker antiferromagnetic coupling, with *J* = −37(1) cm^–1^.^29^ The apparent presence of a monomeric Cr­(II) impurity (∼11%) prevents the magnetic susceptibility from reaching zero at low temperature. The antiferromagnetic coupling in **2** could be attributed to several mechanisms, such as direct exchange via metal–metal bonding and superexchange via the bridging amido ligands. The latter is more likely the larger contributor given the weak metal–metal bonding, as described for complexes with similarly weak metal–metal interactions.^50,51^ The χ_M_ *T* value for **2** increases subtly with increasing temperature, which likely reflects thermal population of higher spin states.^52^ Details of the fitting and magnetic property interpretation are provided in the Experimental Section.

### Quantum Chemical Calculations

DFT calculations were performed for the molecular geometries of both **1** and **2**, and compared with experimental X-ray diffraction data. A comprehensive screening of functionals (see Computational Details) revealed that DFT provides the expected good agreement with experimental values for compound **1**. The Cr–N bond distances showed minimal deviations from reported values, with errors ranging from 0.005 Å for M06 to 0.09 Å for BLYP (Table S4).^53–55^ Among the tested functionals, TPSS-D3 and M06 were the most consistent with experimental values.^56,57^

In contrast to **1**, DFT calculations consistently failed to achieve geometrical accuracy for **2**. The largest discrepancies were observed in the Cr–Cr bond distances, which were systematically underestimated across all tested functionals, with deviations from experiments ranging from 0.391 to 0.523 Å, except for the hybrid functionals B3LYP and TPSSh, which showed overestimation of the Cr–Cr bond by 0.281 and 0.171 Å, respectively (Table S5). The addition of a dispersion correction tended to improve agreement, though not enough to be solely responsible for the large deviations reported. TPSSh-D3 performed best among all DFT methods by more closely reproducing both Cr–Cr bond distances (though a large deviation of 0.094 Å remains for the Cr–Cr distance), as well as other geometric parameters within the diamond core for compound **2** (Table S5). However, this was limited to compound **2**, as TPSSh-D3 exhibited a very large error in the Cr–Cr distance (0.584 Å), comparable to other functionals when applied to compound **3**, and consistent with literature precedent that DFT cannot reliably predict the Cr–Cr bond distance when a bond is present (Table S5 and S6). Additionally, the average Cr–N–Cr angle showed a substantial deviation of up to nearly 18° in most functionals compared with experimental values (68.6°) for **2**.

As mentioned in the Introduction, Cr–Cr bonds are notoriously multireference in nature;^58,59^ therefore, we hypothesized that this interaction could not be captured by conventional exchange-correlation functionals.^25^ Therefore, second-order multireference methods (CASPT2) were employed to study the electronic structure of **2** and **3**. An active space with 10 electrons and 10 orbitals, denoted (10*e*, 10*o*), was selected (Figure 10 and S5). Cr­(II) is a d^4^ metal; therefore, the molecular orbitals consisting of these pairs aligned in phase (“bonding”) and out of phase (“antibonding”) were selected, resulting in 8 electrons in 8 orbitals. An additional pair of ligand–metal bonding/antibonding orbitals was included since prior work has shown such orbitals can lead to improved spin-splitting energies at the CASPT2 level.^60,61^

**Figure 10:**
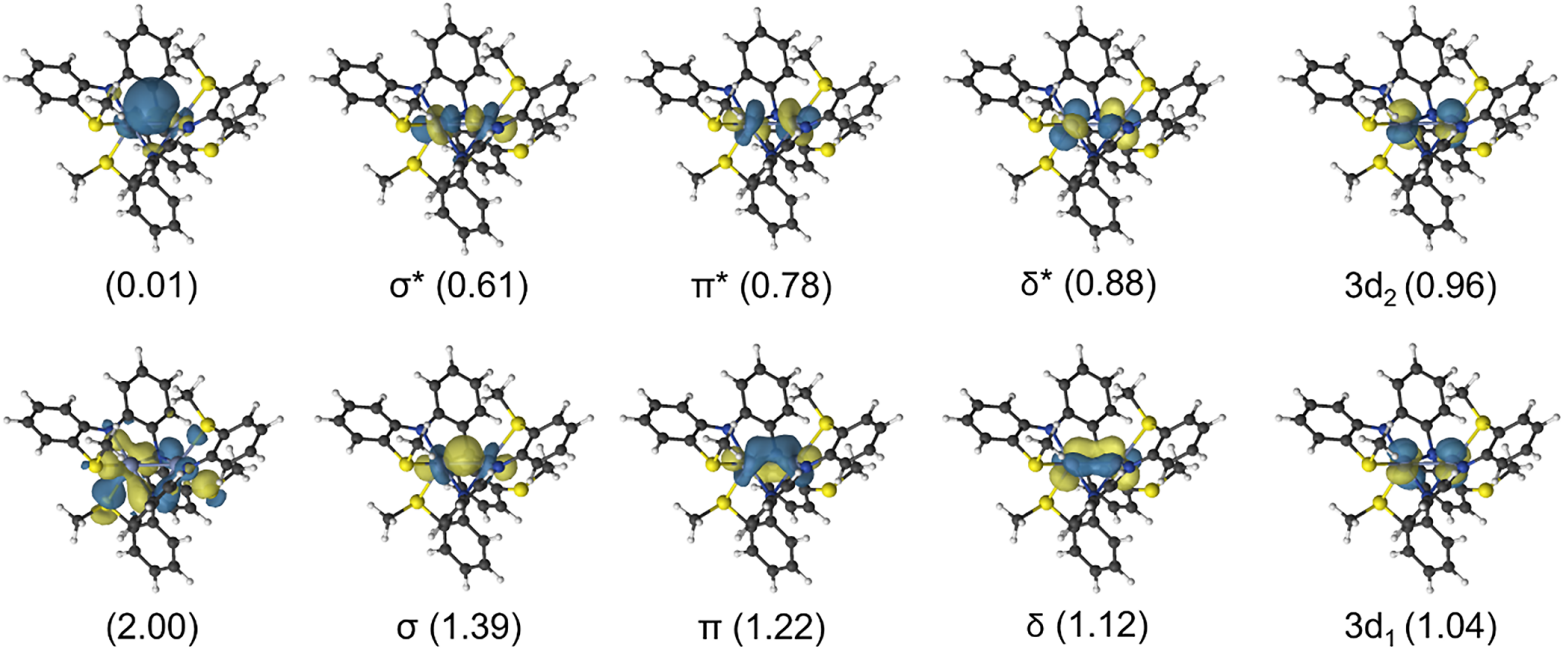
CASSCF active natural orbitals of **2** from the (10*e*, 10*o*) active space computed for the CASPT2 ground state singlet. For simplicity in the discussion, orbitals with sufficient overlap to contribute to a bond are labeled as σ, π, and δ, despite their orientations not matching this assignment perfectly. Analogous active orbitals for **3** are given in Figure S5.

Given our hypothesis that metal–metal interactions are responsible for poor DFT geometries, a scan of the potential energy surface (PES) along the Cr–Cr distance was performed for **2** (Figure S6, Table S7). However, during the constrained optimizations, one of the SMe arms decoordinates when the Cr–Cr distance is varied, presumably due to weak Cr–SMe bonding. In fact, an inversely proportional relationship between the Cr–Cr and Cr–S distances was observed in the scan, indicating that the metal–ligand bonding is quite sensitive to constraints on the metal. Therefore, the first coordination sphere of both metal centers was kept frozen at the positions from XRD during a partial DFT optimization of the remainder of the complex using the PBE functional.^62^ CASPT2 calculations were performed on this experimentally derived structure. We note in passing that the CASPT2 energy obtained was lower by 2.3 kcal/mol than the closest point in the aforementioned scan, supporting the use of this structure for additional discussion.

Of the four potential bonding orbitals, three have significant orbital overlap (Figure 10). For the sake of simplicity in the discussion, they have been labeled as σ, π, and δ, although their orientations do not match this assignment perfectly. The remaining pair of orbitals are assigned as nonbonding and labeled 3d_1_ and 3d_2_. The resulting wave function has significant multiconfigurational character with the leading determinant contributing only 6.7%, making a discussion of a specific single-electron configuration meaningless (Table S11). Therefore, the CASSCF natural orbital occupation numbers (NOON) better represent orbital occupation in the ground-state singlet, and an effective bond order (EBO) can be calculated by,

$$\mathrm{EBO}=\frac{\sum {\mathrm{NOON}}_{\mathrm{bonding}}-\sum {\mathrm{NOON}}_{\mathrm{antibonding}}}{2}$$

The resulting effective bond orders are 0.73 and 0.64 for **2** and **3**, respectively. While three orbital pair sets are involved in the bonding, significant population of the antibonding orbitals reduces the net bond order to that of a single bond rather than a triple bond.

As observed in the experimental structure, each chromium center adopts a distorted square pyramidal coordination geometry when considering the five-coordinate environment. This leads to side-on orbital overlap in which the d orbitals approach each other at an angle and not head-on. The Cr–N_cent_–Cr hinge angle plays a crucial role in determining the extent of d-orbital overlap between metal centers. To explore the influence of orbital overlap, the Cr_1_–N_b_–N_b_–Cr_2_ dihedral angle (which serves to alter the Cr–N_cent_–Cr hinge angle) was varied by 2.5° increments over ±10° from the experimental value (Figure S7). As the dihedral angle is decreased from its minimum value, the d-orbital overlap increases, strengthening the Cr–Cr bond (Table S8). Despite some structural similarities with the dinuclear [Fe_2_(**L2**)_2_] reported previously,^29^ the Fe complex presents a larger dihedral angle (139°) and longer metal–metal distances, consistent with the assignment of no significant Fe–Fe bonding.

Finally, the CASPT2 spin-splitting energies were computed. A ground-state singlet is assigned for both complexes, with a low-lying triplet state falling only 3.2 or 3.0 kcal/mol higher in energy for **2** and **3**, respectively (Table S10). The EBOs in the low-lying triplet states are only slightly smaller compared to the ground-state singlet at 0.66 and 0.57 for complexes **2** and **3**, respectively. This suggests that while the metal–metal bond is weakened in the excited state, it is still present. On the other hand, the quintet states reveal a moderate decrease in the EBO for both compounds, showing 0.46 and 0.44, respectively. This is consistent with the higher energies of the quintet state, falling 11.0 and 8.5 kcal/mol above the ground state for **2** and **3**, respectively, and further weakening of the Cr–Cr bond.

## Discussion

The experimental and theoretical results with **1–3** illuminate factors that account for the differing Cr–Cr distances observed in complexes containing Cr_2_N_2_ diamond cores. They also reveal the nature of the Cr–Cr bond as well as structural and steric contributions that control the nuclearity of these and related chromium complexes.

The structural differences between mononuclear **1** and dinuclear **2** and **3** appear to be attributed to the increased steric profile of the NMe_2_ groups with respect to SMe and OMe. This is consistent with observations made previously in the structures of **L1** and **L2** with Fe­(II). Although dinuclear Fe complexes were isolated with both ligands, a second isomer with **L1** was also isolated without bridging N atoms, again revealing the influence of the increased size of NMe_2_ (Figure 11). It appears that the dinuclear Fe structures can be isolated with **L1** because the longer Fe–N and Fe–Fe distances (i.e., no metal–metal bonds) minimize steric congestion. Consistent with this hypothesis, side-by-side comparisons of the XRD space-filling models for the dinuclear complexes with Cr­(II) (**2**) and Fe­(II) with X = SMe show how the tetradentate ligand is more tightly enveloped around the metals in **2** (Figure 12). We note that the differences in structure with Fe and Cr cannot be attributed simply to changes in the metal ion size. Cr­(II) has a slightly larger ionic radii compared to Fe­(II) in both low- and high-spin configurations.^63^

**Figure 11:**
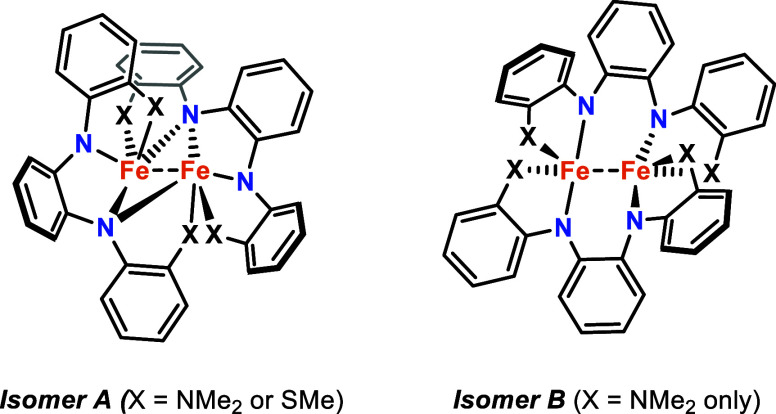
Previously reported isomers of dinuclear Fe­(II) complexes with **L1** and **L2**.

**Figure 12:**
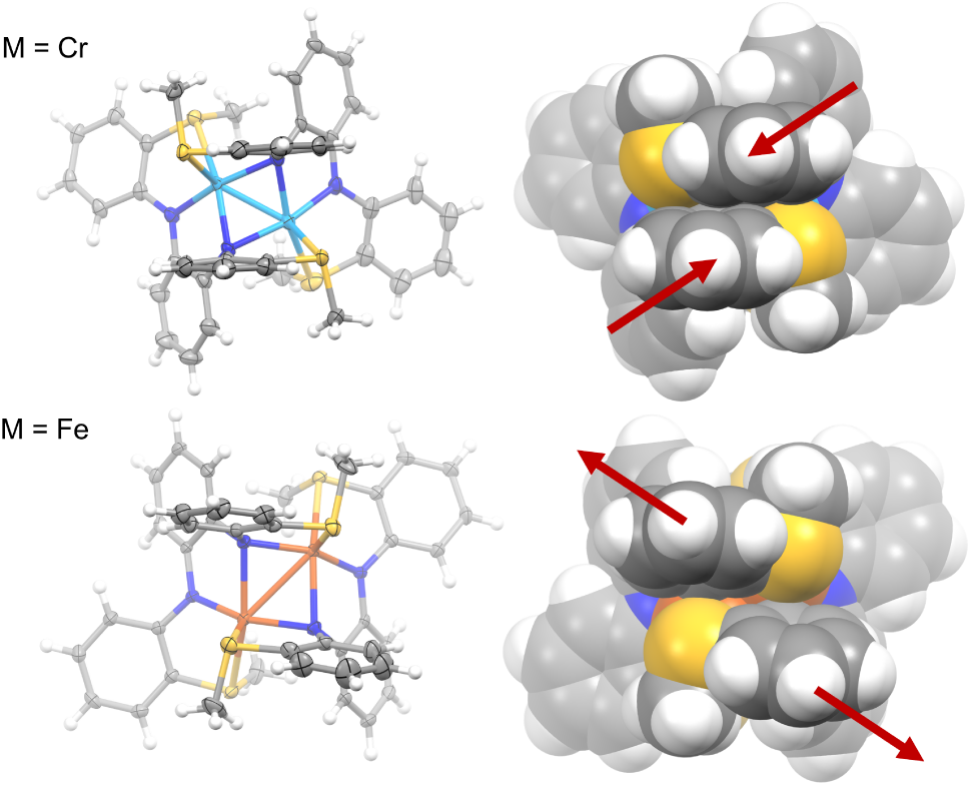
Structural comparison of **2** and the dinuclear Fe­(II) complex containing **L2** with X = SMe. Different enantiomers are shown with Cr (M) and Fe (P). Red arrows were added to emphasize differences in the aryl positioning.

The relatively short Cr–Cr distances in dinuclear **2** and **3** are consistent with Cr–Cr single bonds based on formal shortness ratio calculations of 1.00. CASSCF calculations confirmed the presence of metal–metal bonding (effective bond orders of 0.73 and 0.64, respectively), but the nature of the bond in each complex was revealed to be a net summation of partial overlap between three different sets of Cr 3d orbitals.

With these findings, we can now explain the more general differences in Cr–Cr distances for dinuclear Cr­(II) complexes containing Cr_2_N_2_ diamond cores, as described in the Introduction and summarized in Figure 1. Most Cr_2_N_2_ complexes containing bridging amido ligands have Cr^II^–Cr^II^ distances of ≥2.8 Å. Complexes that fall into this category are almost exclusively those containing monodentate amides that lack additional chelating donor groups. Chelating and rigid multidentate amides like those in **L2** and **L3**, as well as the tridentate (NON)^2–^ ligand in [Cr­[MesN­(SiMe_2_)]_2_O]_2_, force the structures to fold to accommodate both the formation of the Cr_2_N_2_ core and the binding of the different donor groups at coordination sites around each metal.^23^ This folding brings the metals close to each other, which allows three sets of 3d orbitals to undergo side-on overlap for metal–metal bonding, as shown in Figure 13. Betley and coworkers invoked a similar explanation to account for metal-metal orbital overlap in trinuclear complexes containing Fe and Co.^164^

**Figure 13:**
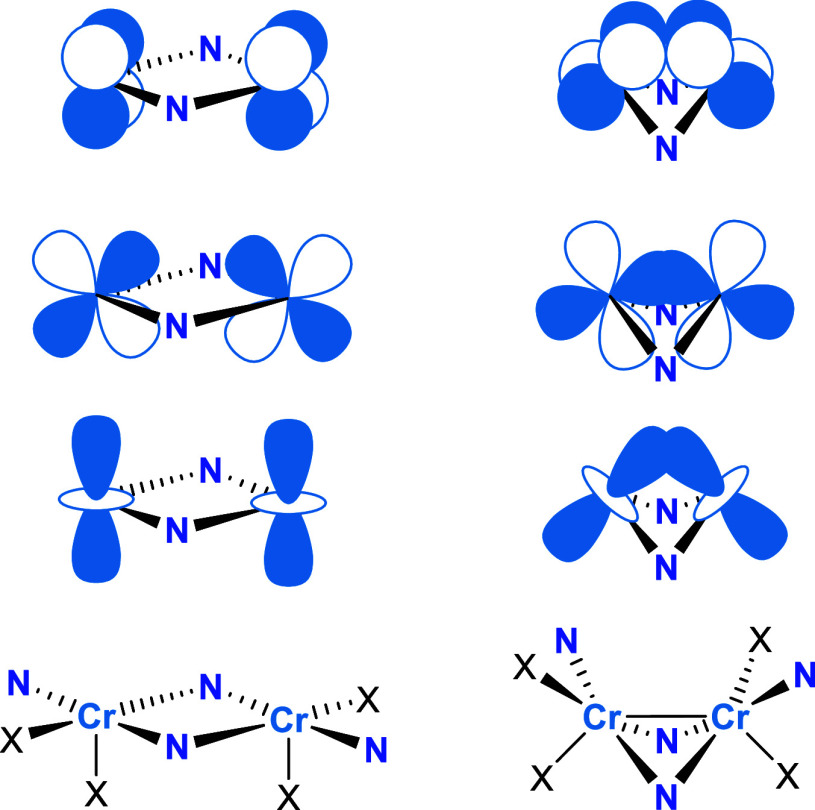
Simplified depiction of how d-orbital overlap changes with ligand-enforced folding along the N···N hinge of the Cr_2_N_2_ core. X = SMe or OMe.

The side-on overlap of 3d orbitals, shown in Figure 13, accounts for the short Cr–Cr distances observed in **2** and **3** at 2.3356(6) and 2.3481(5) Å, and also the 2.384(2) Å Cr–Cr distance reported for [Cr­[MesN­(SiMe_2_)]_2_O]_2_ shown in Figure 1. The Cr–N_cent_–Cr angle—which can be used to define the degree of folding in the Cr_2_N_2_ core—for **2** and **3** is 111.67(7)° and 109.78(7)°, which is nearly identical to that observed in the structure of [Cr­[MesN­(SiMe_2_)]_2_O]_2_ at 109.1(2)°. For comparison, complexes with Cr–Cr distances >2.8 Å tend to have a relatively planar Cr_2_N_2_ core with Cr–N_cent_–Cr angles of 180°. No metal–metal bonding occurs in these latter complexes because the metals are not in the right configuration or proximity for orbital overlap to occur. We also note that the rigid ligand scaffold combined with folding at the bridging amido ligands limits the extent of Cr–Cr bonding that can occur. For example, a recent report from Hernández Sánchez and coworkers showed how a more flexible cyclen-based octamine ligand yielded a dichromium complex with no Cr_2_N_2_ core and a formal metal–metal quadruple bond (*d*_Cr–Cr_ = 1.9609(7) Å).^64^

## Conclusions

In summary, we have shown how variations in the flanking donor groups in triaryl tetradentate ligands derived from *o*-phenylenediamide govern the nuclearity and metal–metal bonding in Cr­(II) complexes. Treating Cr­[N­(SiMe_3_)_2_]_2_(thf)_2_ with H_2_(**L1**) (X = NMe_2_) yielded the mononuclear, square planar complex **1**, whereas the same reactions with H_2_(**L2**) (X = SMe) and H_2_(**L3**) (X = OMe) yielded the dinuclear complexes **2** and **3**. The differences in nuclearity appear to be attributed to the increased steric profile of the NMe_2_ substituents compared to SMe and OMe. Single-crystal XRD structures of **2** and **3** revealed relatively short Cr–Cr distances indicative of single metal–metal bonding. CASSCF calculations confirmed the presence of metal–metal bonding and further revealed that the bonding can be ascribed to partial overlap between three sets of 3d orbitals on Cr. A highly multiconfigurational singlet ground state, with a low-lying triplet state, was assigned via CASPT2.

In addition to nuclearity dependency, chelating amido ligands can be used to enforce structural distortions that orient the metals to facilitate Cr–Cr bonding. The metal–metal bonding is likely weak, as observed for other dichromium complexes with higher formal bond orders,^28^ but the resulting influence on the electronic structure is expected to give rise to differences in reactivity and magnetic properties. Thus, these findings may have implications for the design of first-row transition-metal complexes with reactivity that leverages metal–metal bonds. For example, there are ongoing efforts aimed at using dinucleating ligand scaffolds to configure metal–metal interactions and orient electrostatic fields for cooperative reactions.^65–68^ In this context, complexes **2** and **3** display substituent-dependent differences in reactivity with CH_2_Cl_2_, indicating that the reactivity of these complexes is quite sensitive to subtle ligand modifications. Future efforts will be aimed at further investigating these reactions with dinuclear complexes containing Cr and other first-row metals.

## Experimental Section

All reactions were performed under an atmosphere of N_2_ or Ar using a glovebox or standard Schlenk techniques, unless stated otherwise. Glassware was oven-dried at 150 °C for 12 h and cooled under vacuum or a stream of nitrogen before use. Except for those used for chromatography, solvents were dried and deoxygenated using a Pure Process Technologies Solvent Purification System and stored over 3 Å molecular sieves. Deuterated solvents were deoxygenated by three freeze–pump–thaw cycles and stored over 3 Å molecular sieves. Reagents and chromatography solvents were purchased from commercial vendors and used as received. The N_4_ and N_2_S_2_ proligands H_2_(**L1**) and H_2_(**L2**) were synthesized as described previously.^30,69^ Cr­[N­(SiMe_3_)_2_]_2_(thf)_2_ was prepared using a previously reported procedure.^70^

Infrared spectra were recorded in the range 500–4000 cm^–1^ using a Thermo Scientific Nicolet iS5 with Nujol mulls prepared in a glovebox, except in the case of air-stable products. Raman spectra were collected from crystalline products in sealed capillaries at room temperature using a Renishaw dispersive Raman spectrometer with 514 nm laser excitation. Elemental analysis was carried out with an EAI CE-440 elemental analyzer at the University of Iowa’s MATFab Facility. NMR data were collected on a Bruker AVANCE-300 or DPX-300 operating at 300 MHz. UV–vis spectra were recorded on a Shimadzu UV-1800 spectrophotometer. Measurements were performed in thf using a 1 cm path length quartz cuvette, and baseline correction was applied using thf as a blank.

### H_2_(**L3**)

The N_2_O_2_ proligand H_2_(**L3**) was prepared similarly to H_2_(**L1**) and H_2_(**L2**). Pd_2_(dba)_3_ (0.850 g, 0.928 mmol, 5 mol %), *rac*-BINAP (1.152 g, 1.850 mmol, 10 mol %), NaO^t^Bu (5.280 g, 54.86 mmol), degassed water (1 mL), and *o*-phenylenediamine (2.000 g, 18.36 mmol) were added to toluene (250 mL). The reaction mixture was heated to 105 °C under Ar for 10–20 min, resulting in a bright red solution. Bromoanisole (4.600 mL, 36.94 mmol) was added rapidly by gastight syringe, and the reaction mixture was heated to reflux for 72 h. After cooling to RT, the mixture was treated with a saturated aqueous solution of NH_4_Cl immediately upon exposure to air. The mixture was filtered through a pad of Celite and washed with Et_2_O until the filtrate was colorless. The organic phase was separated, concentrated under reduced pressure, and purified by column chromatography on basic alumina (10:90 ethyl acetate/hexanes) to afford an off-white solid. The solid was recrystallized by the slow evaporation of hexanes and ethyl acetate to yield white crystals. Yield: 3.8 g (64%). HRMS (ESI^+^) *m*/*z* calcd for C_20_H_21_N_2_O_2_ [M + H]^±^ : 321.1598; found: 321.1595 ^1^H NMR (300 MHz, CDCl_3_, δ): 7.34 (dt, *J* = 9.45 Hz, 3.51 Hz, 2H, Ar H), 7.09 (m, 2H, Ar H), 6.96 (q, *J* = 3.51 Hz, 2H, Ar H), 6.85 (m, 6H, Ar H), 6.08 (s, 2H, N–H), 3.84 (s, 6H, CH_3_). ^13^C NMR (300 MHz, CDCl_3_, δ): 55.53 (O–CH_3_), 110.44 (Ar–C), 115.11 (Ar–C), 119.67 (Ar–C), 120.03 (Ar–C), 120.84 (Ar–C), 122.57 (Ar–C), 133.59 (Ar–C), 134.90 (Ar–C), 148.44 (Ar–C).

### Cr­(L1) (**1**)

Freshly prepared Cr­[N­(SiMe_3_)_2_]_2_(thf)_2_ (0.100 g, 0.194 mmol) was treated with a solution of H_2_(**L1**) (0.067 g, 0.19 mmol) in 15 mL of thf. The mixture was stirred overnight, during which time the solution changed from green to dark purple. The next day, the volatiles were removed under vacuum to afford a dark purple solid that was then dissolved in a minimum amount of thf. Filtration of the purple-colored solution through Celite and vapor diffusion with pentane resulted in purple plate-like crystals. Yield: 0.050 g (65%). Anal. Calcd for CrC_22_H_24_N_4:_ C, 66.65; H, 6.10; N, 14.13. Found: C, 66.50; H, 5.99; N, 13.99. IR (cm^–1^): 1550 (m), 1347 (m), 1261 (w), 1203 (w), 1013 (w), 798 (w), 733 (m), 688 (m), 497 (s), 489 (s), 484 (s), 482 (s), 473 (s), 465 (s), 462 (s), 454 (s), 450 (s), 443 (s), 439 (s), 435 (s), 431 (s), 423 (s), 419 (s), 415 (s), 407 (s). Raman (solid, *l* = 540 nm): 364 (w), 472 (w), 501 (w), 579 (w), 649 (m), 731 (w), 863 (m), 895 (w), 1050 (m), 1157 (w), 1201 (w), 1298 (m), 1350 (s), 1393 (m), 1471 (s), 1551 (s), 1585 (s).

### [Cr­(L2)]_2_ (**2**)

To solid Cr­[N­(SiMe_3_)_2_]_2_(thf)_2_, (0.100 g, 0.194 mmol) was added H_2_(**L2**) (0.065 g, 0.19 mmol) dissolved in 15 mL of thf. The mixture was stirred overnight, during which time the solution color changed to dark brown. The next day, the volatiles were removed under vacuum to afford a dark brown solid that was then dissolved in a minimum amount of thf. Filtration of the brown-colored solution through Celite and vapor diffusion with pentane afforded **2** as brown blocks. Yield: 0.086 g (55%). Anal. Calcd for Cr_2_C_40_H_36_N_4_S_4_: C, 59.70; H, 4.47; N, 6.96. Found: C, 59.47; H, 4.63; N, 6.22. IR (cm^–1^) 1568 (w), 1334 (w), 739 (w), 499 (s), 485 (s), 477 (s), 446 (s), 438 (s), 435 (s), 427 (s), 416 (s), 407 (s). Raman (solid, *l* = 540 nm): 285 (w), 303 (w), 348 (w), 433 (m), 587 (w), 657 (w), 708 (w), 858 (s), 1037 (m), 1123 (w), 1155 (m), 1198 (m), 1288 (m), 1314 (m), 1452 (s), 1574 (s).

### [Cr­(L3)]_2_ (**3**)

To solid Cr­[N­(SiMe_3_)_2_]_2_(thf)_2_ (0.100 g, 0.194 mmol) was added H_2_(**L3**) (0.062 g, 0.19 mmol) dissolved in 15 mL of thf. The mixture was stirred overnight, during which time the solution color changed to dark green. The next day, the volatiles were removed under vacuum to afford a dark green solid that was then dissolved in a minimum amount of thf. Filtration of the green-colored solution through Celite and vapor diffusion with pentane resulted in green block-like crystals. Yield: 0.007 g (5%). After isolating **3**, secondary species **4** and **5** were isolated from the mother solution as green needle-like crystals, which were analyzed with single-crystal XRD.

### Crystallographic Studies

Single-crystal XRD data were collected on a Bruker D8 VENTURE DUO diffractometer equipped with an IμS 3.0 microfocus source operated at 75 W (50 kV, 1.5 mA) to generate Mo Kα radiation (λ = 0.71073 Å) and a PHOTON III detector. Crystals were transferred from the vial and placed on a glass slide in type NVH immersion oil by Cargille. A Zeiss Stemi 305 microscope was used to identify a suitable specimen for X-ray diffraction from a representative sample of the material. The crystal and a small amount of oil were collected on a MiTeGen 100 μm MicroLoop and transferred to the instrument, where it was placed under a cold nitrogen stream (Oxford 800 series). The sample was optically centered with the aid of a video camera to ensure that no translations were observed as the crystal was rotated through all of the positions and at various temperatures. A unit cell collection was then carried out. After it was determined that the unit cell was not present in the CCDC database, a data collection strategy was calculated by APEX5.^71^ The crystal was measured for size, morphology, and color.

After data collection, the unit cell was redetermined using a subset of the full data collection. Intensity data were corrected for Lorentz, polarization, and background effects using APEX5.^71^ A numerical absorption correction was applied based on a Gaussian integration over a multifaceted crystal and followed by a semiempirical correction for absorption applied using SADABS.^71^ The program SHELXT^72^ was used for the initial structure solution, and SHELXL^73^ was used for refinement of the structure. Both of these programs were utilized within the OLEX2 software.^74^ Hydrogen atoms bound to carbon atoms were located in the difference Fourier map and geometrically constrained using the appropriate AFIX commands.

### Buried Volume Calculations

UCSF ChimeraX version 1.6.1 was used to calculate the buried volume using the structure files (CIF) from crystallographic coordinates. The SEQCROW plugin was used for the calculation. No specific fit was applied. The buried volume sphere was centered on the metal atom using the experimental coordinates. The calculation follows a fixed-radius method with a default van der Waals radius of 3.5 Å.^40,41^

### Magnetism Studies

Magnetic data for compounds **1** and **2** were collected by using a Quantum Design MPMS3 magnetometer. All sample preparations were performed in a dinitrogen-filled MBraun glovebox. Powdered crystalline samples were loaded into polyethylene bags, sealed, and inserted into drinking straws for measurements. Ferromagnetic impurities were checked through variable-field measurements (0–20 kOe) of the magnetization at 100 K, and linear fits of the *M* vs *H* data indicated the absence of ferromagnetic impurities (Figures S3 and S4). Magnetic susceptibility data were collected between 10 K and 300 K. Data were corrected for the magnetization of the sample holder (plastic bags) by subtracting the susceptibility of an empty plastic bag and for the diamagnetic contributions of the sample by using Pascal’s constants. Fits of magnetic susceptibility data were performed using PHI.^75^

For complex **1**, the best fit obtained from PHI gave *S* = 2, *g* = 1.914(1), a small amount of intermolecular coupling (*zJ*) of −0.4 cm^–1^ (exact fit value −0.386(3) cm^–1^) and substantial temperature-independent paramagnetism (TIP) of 580(20) × 10^–6^ cm^3^/mol. The residual value of the fit was 0.00619. We note that in the best fit, the isotropic *g* value may be negatively correlated with both intermolecular coupling and TIP parameters. Given the overall good agreement with a simple model, we did not add zero-field splitting considerations (*D* and *E*).

For compound **2**, the best fit obtained from PHI for a symmetric dinuclear species (*S*_1_ = *S*_2_ = 2) gives *g*_1_ = *g*_2_ = 1.91(1), antiferromagnetic exchange coupling *J* = −410(3) cm^–1^, and temperature-independent paramagnetism (TIP) of 650 × 10^–6^ cm^3^/mol. To account for the significant nonzero χ_M_ *T* value at low temperature, we include a monomeric *S* = 2 (HS Cr­(II)) impurity set at 11%. The residual value of the fit was 0.000217, and no strong correlations between fit parameters are present. Intriguingly, for compound **2**, a slightly negative value of *M* observed at *H* = 0 initially indicates minimal paramagnetic impurity. It is possible that the experimental *M* vs *H* data suggesting minimal paramagnetic impurity can be consistent with the fit values indicating significant impurity if, for example, the surface behavior of the dinuclear Cr­(II) species in **2** is more like monomeric **1**. Such behavior is noticed in spin-crossover compounds, where bulk and surface magnetic properties differ. Notwithstanding, we note that antiferromagnetic exchange coupling values are not sensitive to the presence of paramagnetic impurities. Given the multiple pathways for magnetic exchange (Cr–Cr bonding, superexchange) and the likely mixing of excited states (large TIP), the true magnetic coupling model is likely more complex than the simplistic model presented here as well as the other models we have tried, but we do not wish to overparametrize fits to the magnetic susceptibility. The values quoted in the results section should be treated provisionally, but nevertheless are qualitatively different from the dinuclear Fe analogue.

### Computational Studies

DFT calculations were carried out using Turbomole V7.3 with a wide range of functionals such as PBE, PBE0, TPSS, TPSSh, BLYP, B3LYP, M06, and M06-L.^53–56,62,76–80^ With the exception of M06 and M06-L, the impact of dispersion corrections has also been studied within each functional by applying Grimme’s D3 correction.^57^ The def2-TZVP basis set was employed for all atoms. The resolution-of-identity (RI) approximation was employed for the Coulomb integrals, and an SCF convergence criteria of 1.0 × 10^–7^ a.u. was used.^81^ Geometry optimizations and harmonic vibrational frequency calculations were carried out in the quintet spin state for **1** and the singlet spin state for **2** and **3**. The Cartesian gradient was converged to 1.0 × 10^–4^. Given the structural flexibility in the Cr_2_N_2_ core and the presence of a Cr–Cr bond, full geometry optimizations were in poor agreement with solid-state measurements regardless of functional choice; therefore, a second set of geometry optimizations were performed with the PBE functional in which the metal centers and the atoms in the first coordination sphere were kept fixed at the positions determined from diffraction resulting in the so-called “experimentally derived” structures.

To confirm the presence of a chromium–chromium bond in **2** and **3**, complete active space calculations (CASSCF) were performed on their experimentally derived structures.^87^ The active space described earlier is ten electrons in ten orbitals, (10*e*, 10*o*). The all-electron ANO-RCC basis sets of triple-ζ quality were used on the metal centers and atoms in the first coordination sphere, but a smaller basis set was used for peripheral atoms.^82–84^ Specifically, the following contractions were used: 6s5p3d2f1g for Cr, 4s3p2d1f for N, 5s4p2d1f for S, 4s3p2d1f for O, 2s1p for C, and 1s for H. Moreover, relativistic effects were included via the scalar second-order Douglas–Kroll–Hess Hamiltonian. The CASSCF energy was converged to a threshold of 1.0 × 10^–7^ a.u.

To assign a ground state, second-order complete active space multireference computations (CASPT2) were performed.^88,89^ The singlet to the nonet spin states were computed for **2** and **3**. The zeroth order Hamiltonian was computed using an imaginary shift of 0.2 a.u. to avoid intruder states along with the default value of the IPEA shift of 0.25.^90^ Cholesky decomposition was employed in addition to local exchange screening to facilitate integral evaluation.^91^ The CASPT2 energy was converged to a threshold of 1.0 × 10^–7^ a.u. CASSCF and CASPT2 calculations were performed as implemented in the OpenMolcas Software package V24.02.^92^ Finally, a scan of the CASPT2 potential energy surface along the hinge angle was performed for **2** in the *S* = 0 and *S* = 1 states (see Table S7 for structures associated with the scan).

## Supplementary Material





## Data Availability

A collection of computational results including XYZ files of optimized geometries, input, and output files is available in a FigShare repository: https://doi.org/10.6084/m9.figshare.30999682.

## Notes

Optimized geometries from calculations (ZIP)
Tabulated crystallographic data and structures of H_2_(**L3**) and **5**; SQUID magnetometry data; tabulated data and plots from DFT and CASSCF calculations; experimental spectra (PDF)
