# Supplementary material for: Leveraging Chelating Amido Ligands to Support Metal–Metal Bonding in Dinuclear Cr(II) Complexes
Source: Inorg Chem. 2026 Mar 5;65(10):5850–63. doi: 10.1021/acs.inorgchem.6c00424 (PMC12997166; doi:10.1021/acs.inorgchem.6c00424)
Supplement: Supplementary file 2 [file ic6c00424_si_002.pdf]

Supporting Information

Leveraging Chelating Amido Ligands to Support Metal-Metal Bonding in Dinuclear Cr(II) Complexes

Janadhi L. Ananda Nakath Durage,[a] Joan Cardona,[a,b] Soumen Sinhababu,[c] Darby H. Duffy,[a] Daniel Martinez,[a] Matthew P. Shores,[c] Daniel K. Unruh,[a,d] Bess Vlaisavljevich,[a]* Scott R. Daly[a]*

[a]Department of Chemistry, The University of Iowa, Iowa City, Iowa 52442, United States

[b]Departament de Química Inorgànica i Orgànica and Institut de Recerca de Química Teòrica i Computacional, Universitat de Barcelona, Diagonal 645, 08028 Barcelona, Spain

[c]Department of Chemistry, Colorado State University, Fort Collins, Colorado 80523, United States

[d]The University of Iowa, Office of the Vice President for Research, 2660 UCC, Iowa City, Iowa 52242, United States

Email: scott-daly@uiowa.edu, bess-vlaisavljevich@uiowa.edu

Table of contents

1. Crystallographic Details 2

2. Magnetism Data 5

3. Computational Data 6

4. NMR Spectra of **L3** 12

5. FT-IR Spectra 14

6. Raman Spectra 17

7. UV-vis-NIR Spectra 18

8. ^{1}H NMR Spectra of **1** – **3** 21

1. Crystallographic Details

Table S1. Crystallographic data for H_2(**L3**).

**Chemical formula**	**$C_{20}H_{20}N_2O_2$**
**Identifier**	dal25_174
**FW (g mol-1)**	320.38
**Crystal system**	triclinic
**Space Group**	P-1
**a (Å)**	8.0002 (9)
**b (Å)**	13.507 (3)
**c (Å)**	15.965(4)
**α (deg)**	92.142(7)
**β (deg)**	94.048(10)
**γ (deg)**	104.646(4)
**volume $(Å)^3$**	1662.2 (6)
**Z**	4
**ρcalc (g cm^{-3})**	1.280
**μ (mm^{-1})**	0.083
**F (000)**	680.0
**2θ range (deg)**	3.924 to 56.776
**R (int)**	0.0482
**data/restraints/parameter s**	8326/0/454
**GOF**	1.068
**R_1 [$I > 2\sigma(I)$]^a**	0.0451
**wR_2 (all data)^b**	0.1128
**Largest Peak/Hole (e · $Å^{-3}$)**	0.336/-0.218

Table S2. Crystallographic data for **1** – **5**.

**Complex**	**1**	**2**	**3**	**4**	**5**
**Chemical formula**	$C_{22}H_{24}CrN_4$	$C_{40}H_{36}Cr_2N_2S_4$	$C_{40}H_{36}Cr_2N_4O_4$	$C_{94}H_{100}Cl_2Cr_4N_8O_{11.5}$	$C_{85}H_{84}ClCr_4N_8O_9$
**Identifier**	dal24_31	dal24_18	dal24_62	dal24_68	dal24_65
**FW (g mol-1)**	396.45	805.00	740.73	1804.71	1532.90
**Crystal system**	Orthorhombic	Monoclinic	Monoclinic	Monoclinic	Orthorhombic
**Space Group**	Pnma	$P2_1/c$	$P2_1/c$	C2/c	Aea2
**a (Å)**	14.8360 (5)	11.5039 (3)	15.3250 (6)	45.5074 (13)	24.8421 (9)
**b (Å)**	20.7880 (6)	12.2858 (4)	12.3764 (5)	11.1258 (3)	29.4495 (13)
**c (Å)**	6.1369 (2)	28.3911 (9)	17.7436 (7)	37.8771 (11)	11.0258 (4)
**α (deg)**	90	90	90	90	90
**β (deg)**	90	100.4990 (10)	96.951 (2)	121.8460 (10)	90
**γ (deg)**	90	90	90	90	90
**volume $(Å)^3$**	1892.69 (10)	3945.5(2)	3340.7 (2)	16290.6 (8)	8066.3 (5)
**Z**	4	4	4	8	4
**ρcalc (g cm^{-3})**	1.391	1.416	1.473	1.472	1.262
**μ (mm^{-1})**	0.618	0.799	0.701	0.656	0.616
**F (000)**	832.0	1748.0	1536.0	7536.0	3172.0
**2θ range (deg)**	5.492 to 56.578	4.202 to 56.646	4.022 to 56.656	3.81 to 54.968	4.272 to 51.364
**R (int)**	0.0444	0.0463	0.0731	0.0586	0.0669
**data/restraints/ parameters**	2408/0/126	9823/733/592	8321/0/455	18706/0/972	7654/205/503
**GOF**	1.051	1.156	1.032	1.036	1.085
**R_1 [$I > 2σ(I)$]^a**	0.0290	0.0422	0.0343	0.0431	0.0471
**wR_2 (all data)^b**	0.0898	0.0948	0.0897	0.1262	0.1170
**Largest Peak/Hole (e · $Å^{-3}$)**	0.23/-0.31	0.49/-0.43	0.37/-0.55	0.59/-0.64	0.33/-0.36

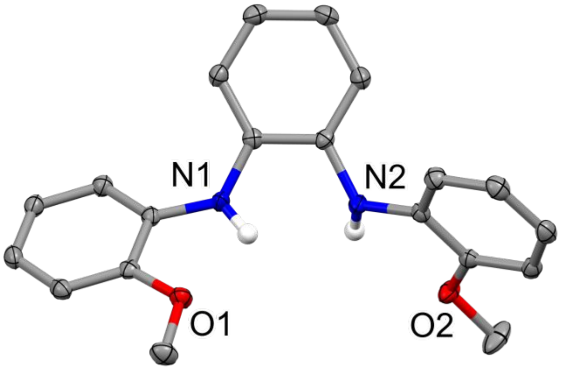

Figure S1. Molecular structure of H_2(**L3**). Ellipsoids for non-carbon atoms are drawn at 50% probability. Carbon atoms are drawn as capped sticks. Hydrogen atoms, disordered components, and the second molecule of H_2(**L3**) in the unit cell have been omitted.

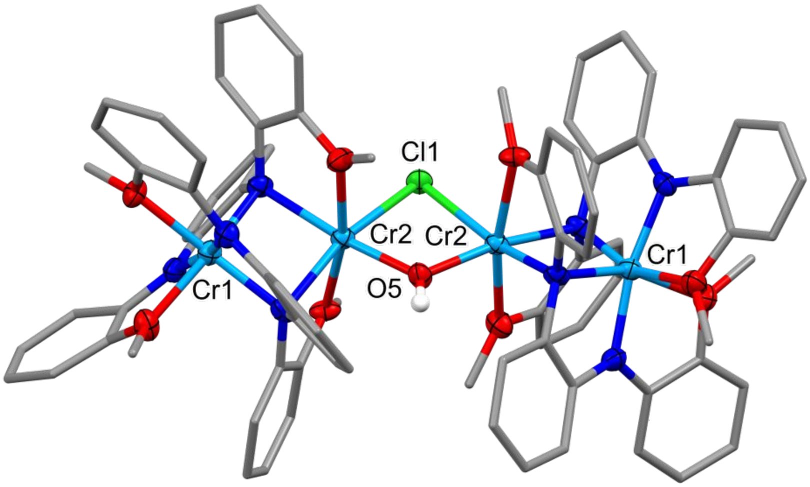

Figure S2. Molecular structure of **5**. Ellipsoids for non-carbon atoms are drawn at 50% probability. Carbon atoms are drawn as capped sticks. Hydrogen atoms and disordered components have been omitted.

2. Magnetism Data

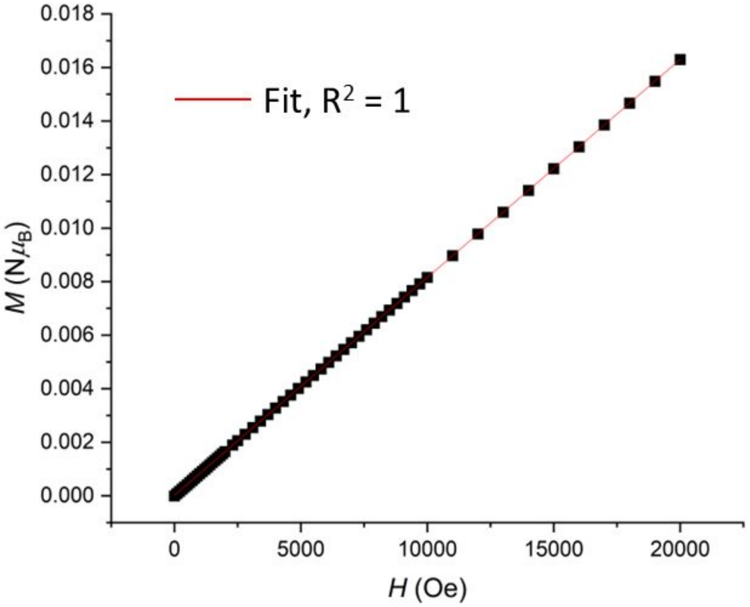

Figure S3. Field dependence of magnetization for **1** collected at 100 K.

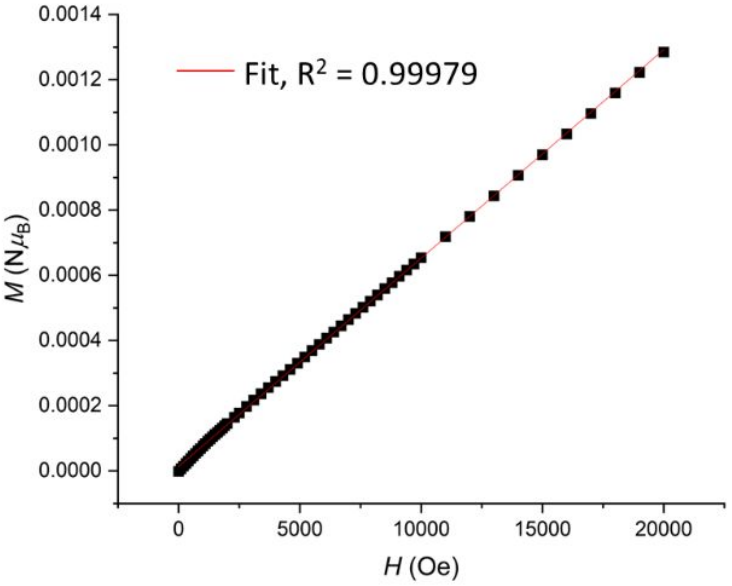

Figure S4. Field dependence of magnetization for **2** collected at 100 K.

3. Computational Data

Table S4. Computed Cr-N distances of compound **1**. Absolute deviations from experiment in parentheses.

Functional	Cr-N_1 (Å)	Cr-N_2 (Å)
**PBE-D3**	1.952(0.018)	2.201(0.038)
**PBE**	1.955(0.015)	2.210(0.047)
**PBE0-D3**	1.952(0.018)	2.195(0.032)
**PBE0**	1.954(0.016)	2.204(0.041)
**TPSS-D3**	1.953(0.017)	2.190(0.027)
**TPSS**	1.956(0.014)	2.202(0.039)
**TPSSh-D3**	1.953(0.017)	2.190(0.027)
**TPSSh**	1.956(0.014)	2.201(0.038)
**M06**	1.954(0.016)	2.168(0.005)
**M06L**	1.959(0.012)	2.185(0.022)
**B3LYP-D3**	1.962(0.008)	2.220(0.057)
**B3LYP**	1.965(0.005)	2.234(0.071)
**BLYP-D3**	1.963(0.008)	2.220(0.057)
**BLYP**	1.969(0.001)	2.249(0.086)
**Experimental**	**1.970**	**2.163**

Table S5. Computed geometrical features of compound **2**. Absolute deviations from experiment in parentheses.

**Functional**	**Cr-Cr (Å)**	**Cr-N-Cr (°)**	**Cr-N$_{1.4}$ (Å)**	**Cr-N$_{2.3}$ (Å)**	**Cr-S$_{1.4}$ (Å)**	**Cr-S$_{2.3}$ (Å)**
**PBE-D3**	1.840(0.496)	51.9(16.7)	1.965(0.031)	2.103(0.030)	2.393(0.028)	2.604(0.013)
**PBE**	1.846(0.490)	51.9(16.7)	1.971(0.025)	2.087(0.014)	2.406(0.015)	2.621(0.004)
**PBE0-D3**	1.869(0.467)	52.7(15.9)	1.945(0.051)	2.103(0.030)	2.416(0.005)	2.533(0.084)
**PBE0**	1.945(0.391)	55.7(12.9)	1.939(0.057)	2.079(0.006)	2.424(0.003)	2.473(0.145)
**TPSS-D3**	1.842(0.494)	52.2(16.4)	1.959(0.037)	2.096(0.023)	2.395(0.026)	2.586(0.031)
**TPSS**	1.857(0.479)	52.4(16.2)	1.966(0.30)	2.102(0.029)	2.414(0.007)	2.596(0.021)
**TPSSh-D3**	2.430(0.094)	71.2(2.6)	1.990(0.006)	2.088(0.015)	2.435(0.014)	2.584(0.033)
**TPSSh**	2.507(0.171)	73.2(4.6)	2.000(0.004)	2.103(0.030)	2.465(0.044)	2.616(0.001)
**[a]M06**	-	-	-	-	-	-
**M06L**	1.813(0.523)	51.0(17.6)	1.971(0.026)	2.106(0.033)	2.437(0.016)	2.679(0.061)
**B3LYP-D3**	2.535(0.199)	73.7(5.1)	2.006(0.010)	2.114(0.041)	2.474(0.053)	2.652(0.035)
**B3LYP**	2.617(0.281)	75.7(7.1)	1.994(0.002)	2.131(0.058)	2.470(0.049)	2.454(0.164)
**BLYP-D3**	1.834(0.502)	51.2(17.4)	1.982(0.014)	2.123(0.050)	2.433(0.012)	2.675(0.058)
**BLYP**	1.846(0.490)	51.2(17.4)	1.995(0.001)	2.137(0.064)	2.468(0.047)	2.734(0.117)
**Experimental**	**2.336**	**68.6**	**1.996**	**2.073**	**2.421**	**2.618**

[a]M06 did not converge to a minima.

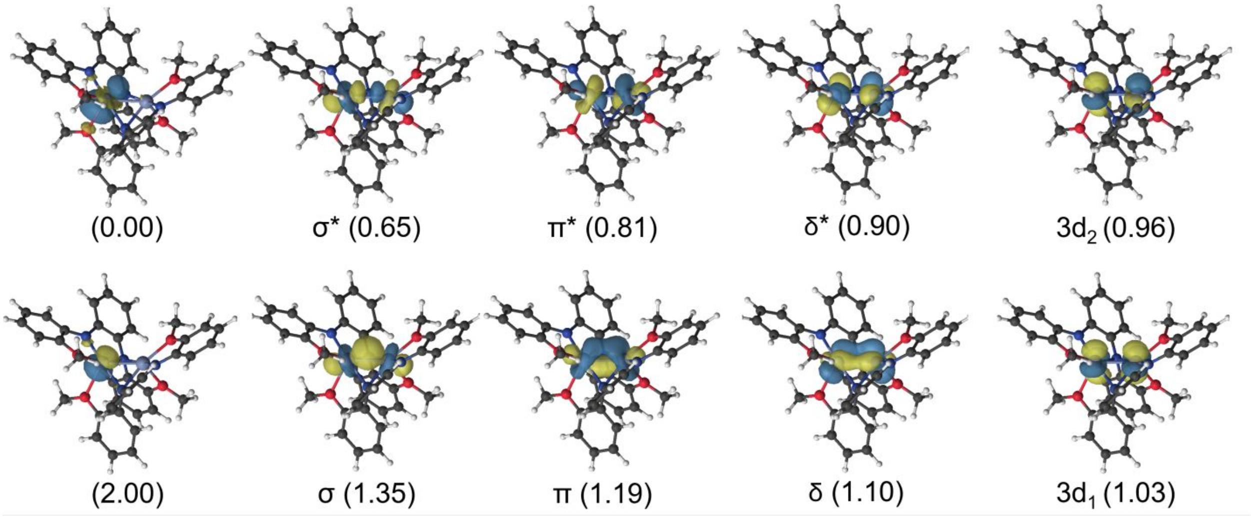

Figure S5. CASSCF active natural orbitals of **3** from the (10*e*, 10*o*) active space computed for the CASPT2 ground state singlet. For simplicity in the discussion, orbitals with sufficient overlap to contribute to a bond are labeled as σ, π, and δ despite their orientations not matching this assignment perfectly.

Table S6. Computed geometrical features of compound **2** using best performing methods from Table S1. Absolute deviations from experiment in parentheses.

**Functional**	**Cr-Cr (Å)**	**Cr-N-Cr (°)**	**Cr-N$_{1.4}$ (Å)**	**Cr-N$_{2.3}$ (Å)**	**Cr-S$_{1.4}$ (Å)**	**Cr-S$_{2.3}$ (Å)**
**TPSSh-D3**	1.765(0.584)	64.90(3.6)	1.952(0.024)	2.083(0.001)	2.112(0.014)	2.337(0.013)
**B3LYP-D3**	1.751(0.587)	65.40(3.1)	1.956(0.020)	2.100(0.015)	2.142(0.045)	2.377(0.053)
**Experimental**	**2.348**	**68.550**	**1.976**	**2.084**	**2.097**	**2.324**

Table S7. CASPT2 energies and geometrical features from Cr-Cr bond scan of compound **2**.

**Cr-Cr (Å)**	**Energy (ha)**	**Cr-N-Cr (°)**	**Cr-N$_{1.4}$ (Å)**	**Cr-N$_{2.3}$ (Å)**	**Cr-S$_{1.4}$ (Å)**	**Cr-S$_{2.3}$ (Å)**
2.636	-5444.122679	76.4	1.916	2.131	2.356	2.262
2.536	-5444.132090	73.3	1.915	2.123	2.363	2.286
2.436	-5444.141307	70.2	1.917	2.116	2.371	2.315
**2.336**	**-5444.150334**	**67.1**	**1.922**	**2.111**	**2.378**	**2.349**
2.236	-5444.159083	64.1	1.931	2.106	2.384	2.388
2.136	-5444.167278	61.0	1.941	2.102	2.390	2.434
2.036	-5444.174388	57.9	1.951	2.101	2.395	2.489

Table S8. CASSCF Natural orbital occupation numbers (NOON) for Cr_2 metal centers 3d orbitals from the diherdal angle scan of compound **2**.

**Geom. Feat.**	**Orbital**							
**Cr-N$_2$-N$_3$-Cr (°)**	**σ**	**σ***	**π**	**π***	**δ**	**δ***	**3d$_1$**	**3d$_2$**
104.1	1.46	0.54	1.30	0.70	1.18	0.82	1.06	0.94
106.6	1.44	0.56	1.27	0.73	1.16	0.84	1.05	0.95
109.1	1.41	0.59	1.24	0.76	1.13	0.87	1.05	0.95
**111.6**	**1.39**	**0.61**	**1.22**	**0.78**	**1.12**	**0.88**	**1.04**	**0.96**
114.1	1.36	0.64	1.20	0.80	1.10	0.90	1.04	0.96
116.6	1.34	0.66	1.18	0.82	1.08	0.92	1.03	0.97
119.1	1.32	0.68	1.16	0.84	1.07	0.93	1.03	0.97
121.6	1.30	0.70	1.14	0.86	1.06	0.94	1.03	0.97

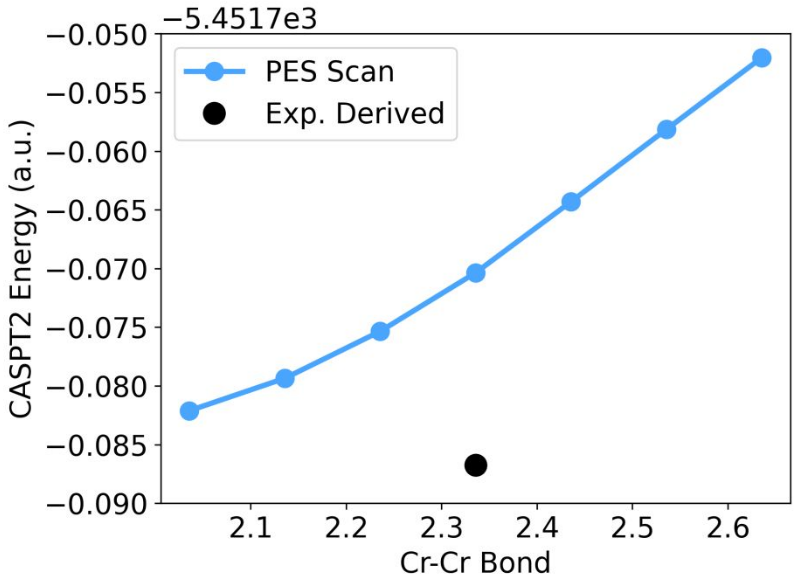

Figure S6. CASPT2 energies and geometrical features from Cr-Cr bond scan of compound **2**. The experimentally derived structure (first coordination sphere frozen in a constrained optimization) is plotted in black.

Table S9. CASPT2 energies and Cr-Cr distance from the dihedral angle scan of compound **2**.

Cr-N_2-N_3-Cr (°)	Energy (ha)	Cr-Cr (Å)
104.1	-5451.783148	2.227
106.6	-5451.785111	2.264
109.1	-5451.786601	2.301
**111.6**	**-5451.786724**	**2.336**
114.1	-5451.787473	2.370
116.6	-5451.786489	2.403
119.1	-5451.78546	2.434
121.6	-5451.783460	2.465

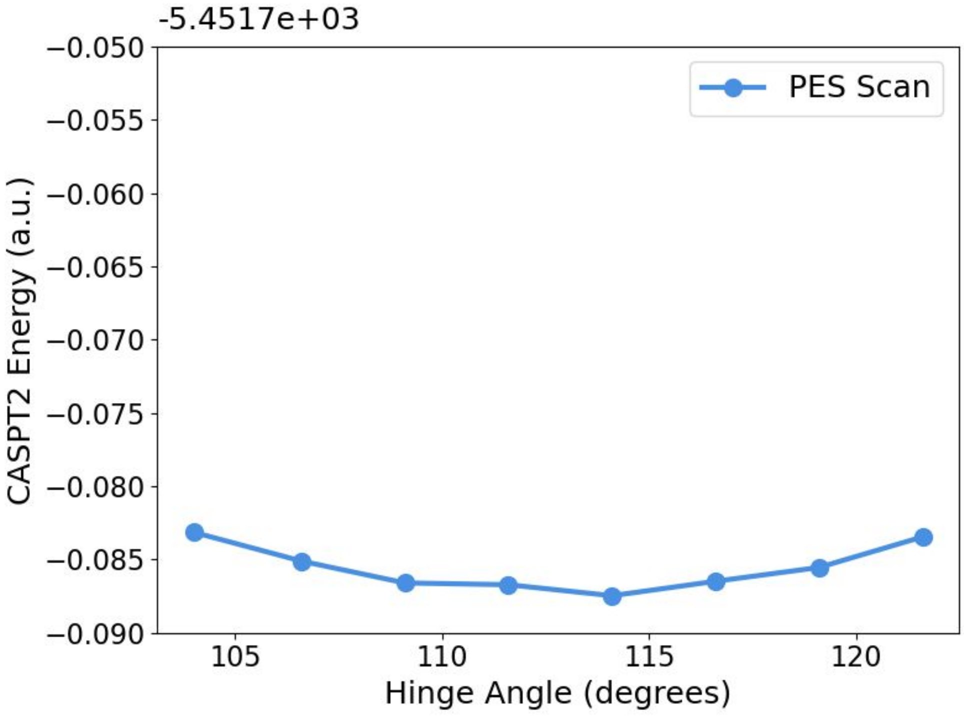

Figure S7. CASPT2 energies and geometrical features from the scan of the hinge angle in compound **2**. The y axis is kept that the same range as Figure S6 to emphasize that the surface along the angle is very flat changing by 2.7 kcal/mol at the highest point.

Table S10. Spin splitting energies for different spin states computed on the PBE/def2-TZVP optimized geometry of compounds **2** and **3**.

	**2**		**3**	
**Spin State**	**Energy (ha)**	**ΔE (kcal/mol)**	**Energy (ha)**	**ΔE (kcal/mol)**
Nonet	-5451.733269	33.6	-4157.094341	27.8
Septet	-5451.754397	20.3	-4157.111906	16.8
Quintet	-5451.769182	11.0	-4157.125096	8.5
Triplet	-5451.781684	3.2	-4157.133978	3.0
Singlet	-5451.786740	0.0	-4157.138710	0.0

Table S11. CASSCF wavefunction for singlet experimentally derived structure of **2.**

**CASSCF Wavefunction (L)(δ*)(δ)(σ)(π*)(σ*)(3d$_2$)(π)(3d$_1$)(L*)**	**Coefficient**	**Weight (%)**
2202202000	-0.13387	1.8
2202200020	0.14165	2.0
2022202000	0.15953	2.5
2022200020	-0.1707	2.9
2uu220d0d0	-0.1714	2.9
2202u0dud0	0.15978	2.6
2022u0dud0	-0.19602	3.8
2022u0udd0	-0.10939	1.2
2uu2d02d00	-0.19416	3.8
2uu2d00d20	-0.20868	4.4
220u2ud0d0	0.11116	1.2
202u2ud0d0	-0.12693	1.6
2uud2d2000	0.11256	1.3
2uud2d0020	-0.11799	1.4
2202002200	0.19141	3.7
2202000220	-0.20623	4.3
2022002200	-0.23897	5.7
**2022000220**	0.2604	6.8
202udu2d00	0.1068	1.1
202udu0d20	0.11394	1.3
2uu200d2d0	-0.25313	6.4
2uududdud0	0.11442	1.3
2uuudu ddd0	-0.12672	1.6
2uu022d0d0	0.10682	1.1
220u0ud2d0	-0.14732	2.2
202u0ud2d0	0.17685	3.1
2uud0d2200	0.15144	2.3
2uud0d0220	-0.16188	2.6
2020u2dud0	-0.10466	1.1
2uu0d22d00	-0.11102	1.2
2uu0d20d20	-0.11537	1.3
2200022200	-0.10531	1.1
2200020220	0.10995	1.2
2020022200	0.11833	1.4
2020020220	-0.1251	1.6
2uu002d2d0	0.12997	1.7

4. NMR Spectra of L3

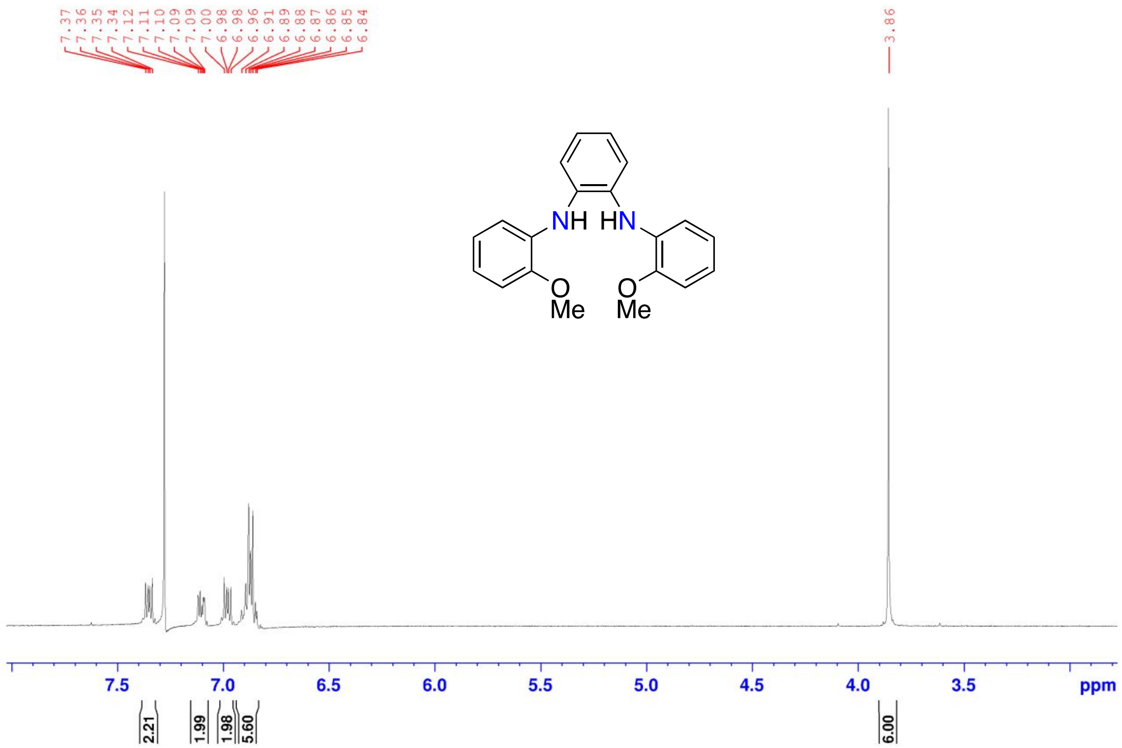

Figure S8. ^1H NMR spectrum of H_2(**L3**) (X = OMe) in $CDCl_3$.

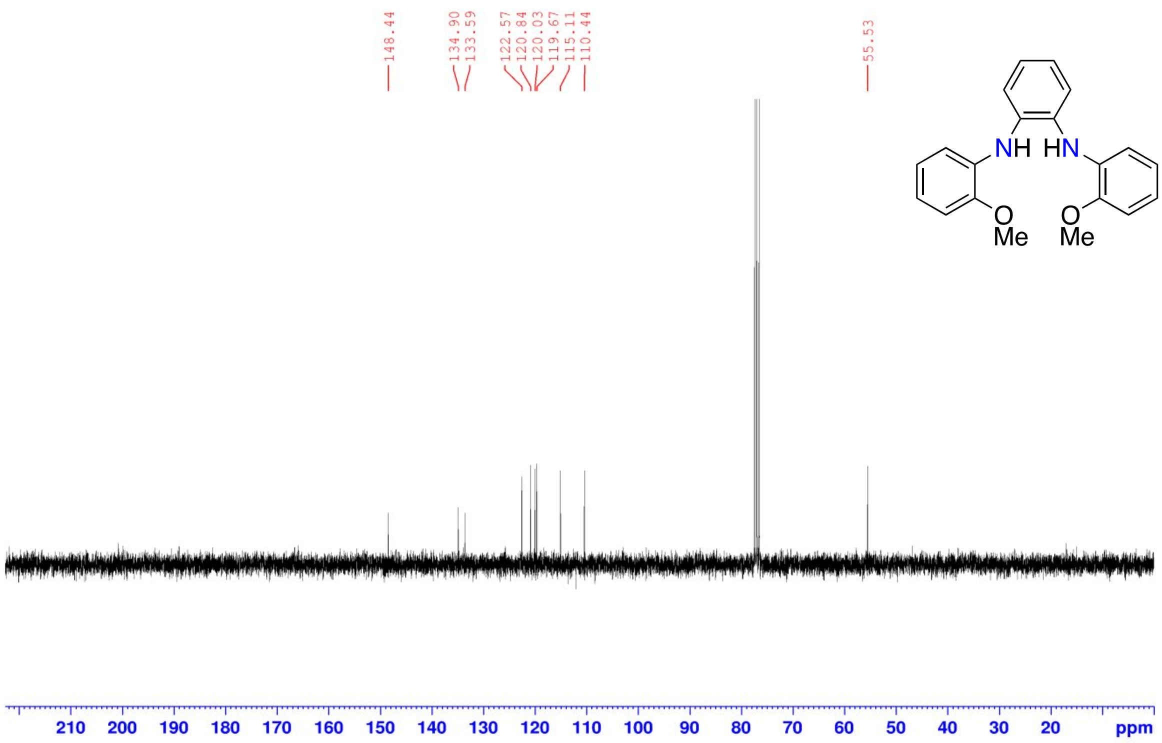

Figure S9. ^{13}C NMR spectrum of H_2(**L3**) (X = OMe) in $CDCl_3$.

5. FT-IR Spectra

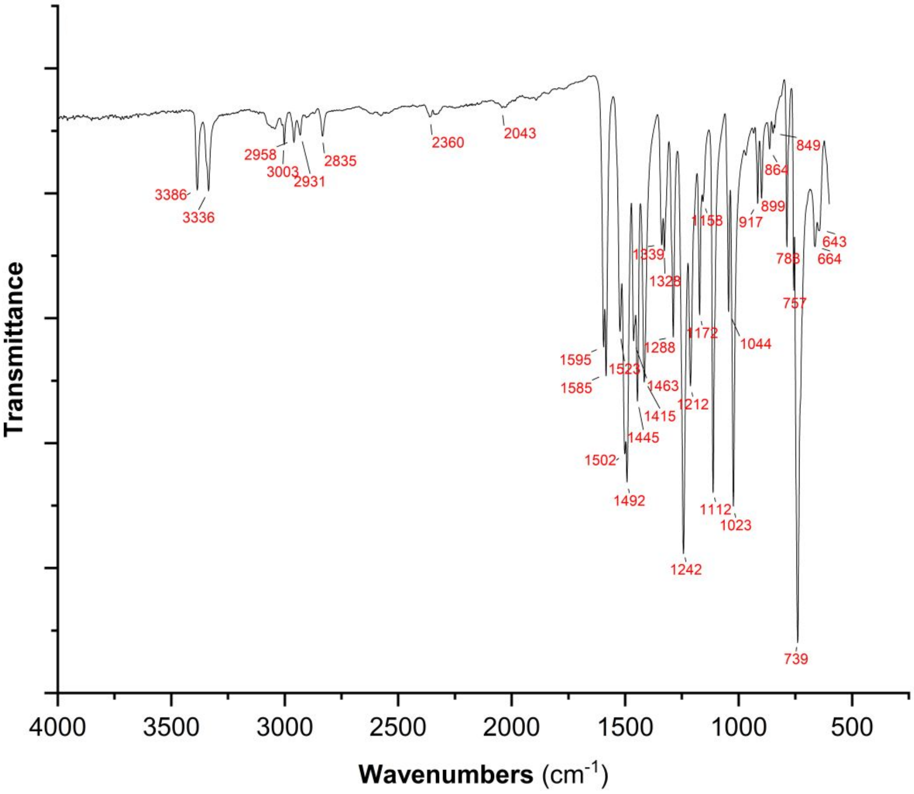

Figure S10. ATR-IR spectrum of H_2(**L3**) (X = OMe).

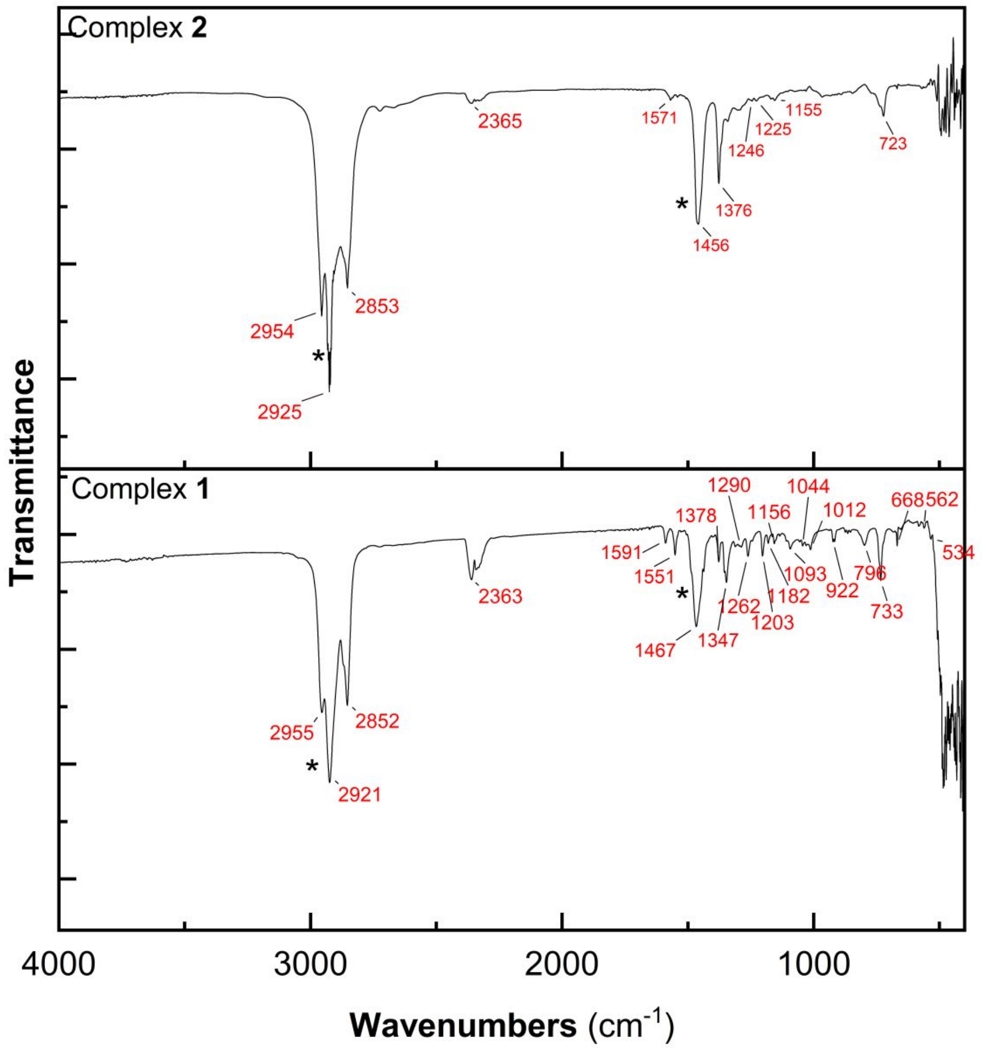

Figure S11. IR spectra (Nujol) of Cr(**L1**) (**1**) and $[Cr(\mathbf{L2})]_2$ (**2**). Peaks marked with * are assigned to Nujol. The features around 2365 cm^{-1} are CO_2 from the background.

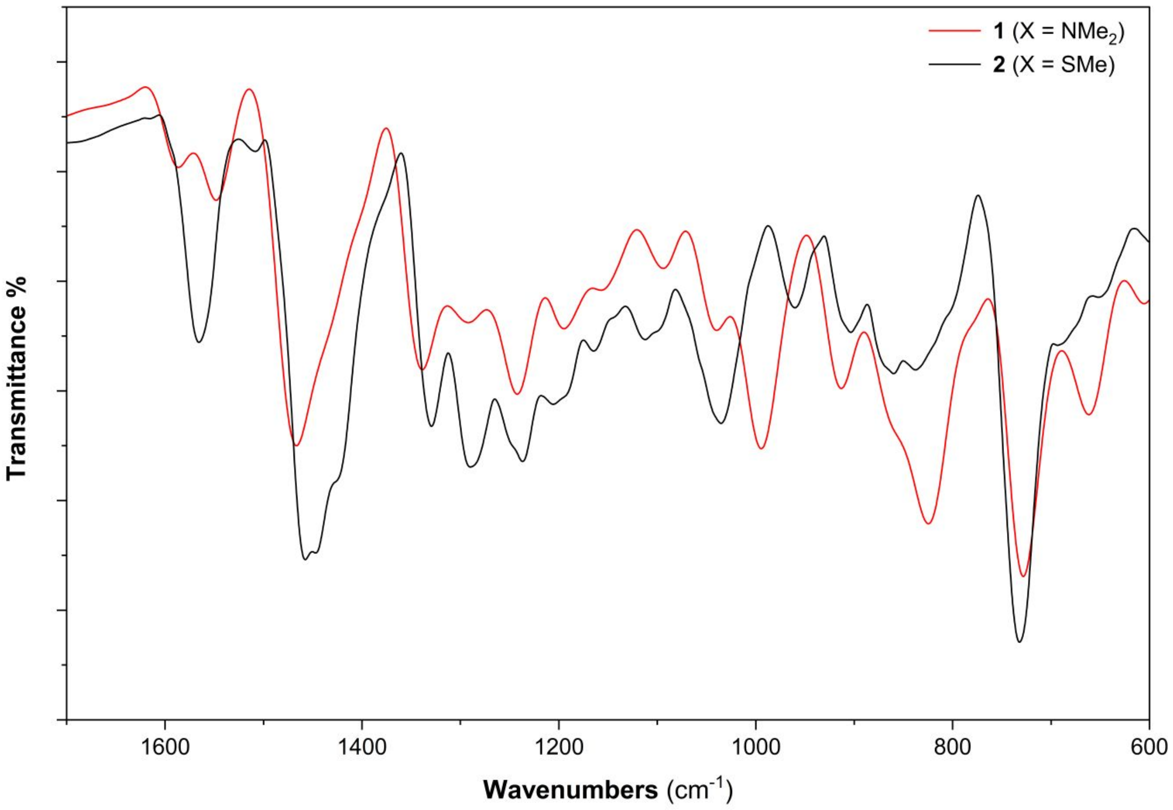

Figure S12. Overlay of ATR spectra (fingerprint region) of Cr(**L1**) (**1**) and [Cr(**L2**)]$_2$ (**2**).

6. Raman Spectra

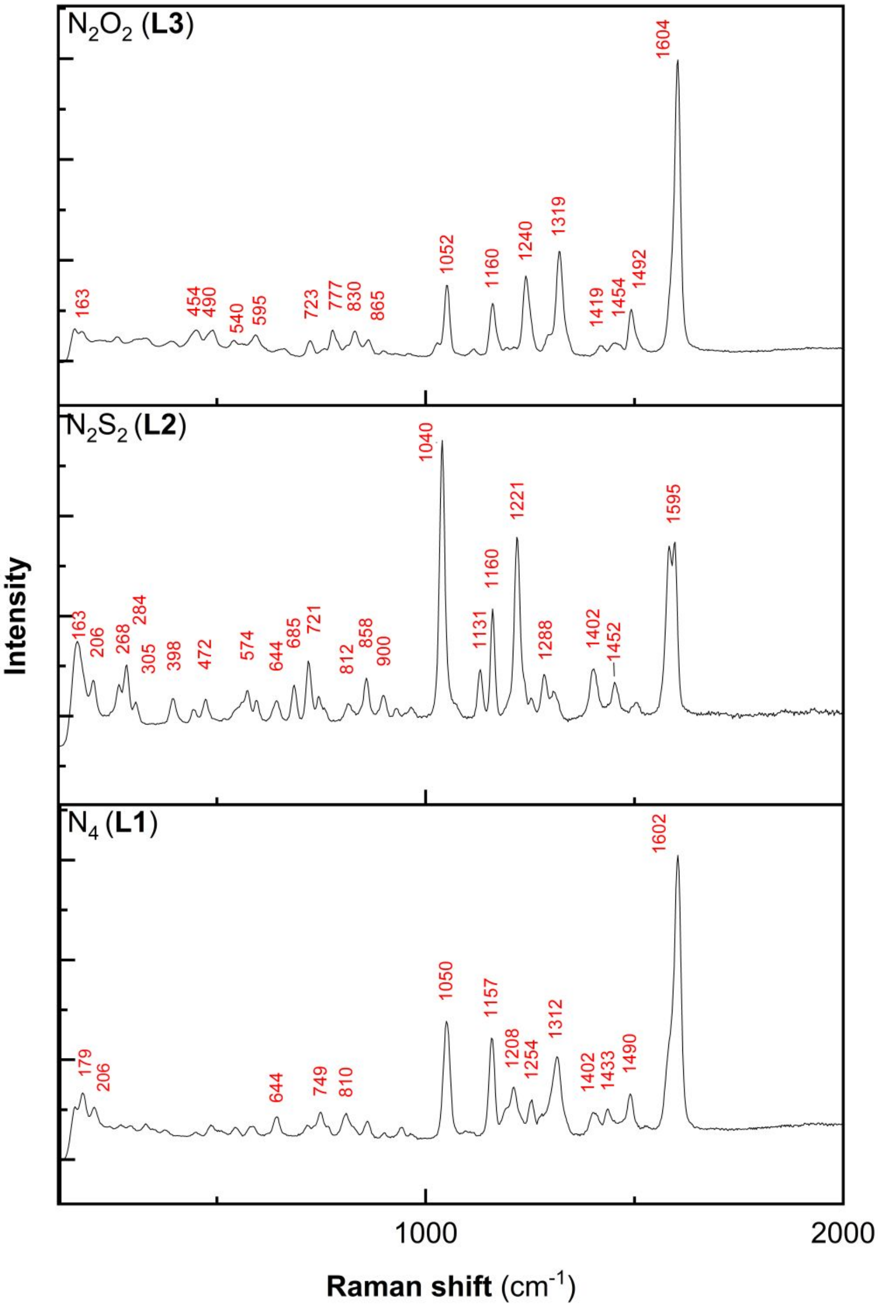

Figure S13. Solid-state Raman spectra of H_2(**L1**) (X = NMe_2), H_2(**L2**) (X = SMe), and H_2(**L3**) (X = OMe).

7. UV-vis-NIR Spectra

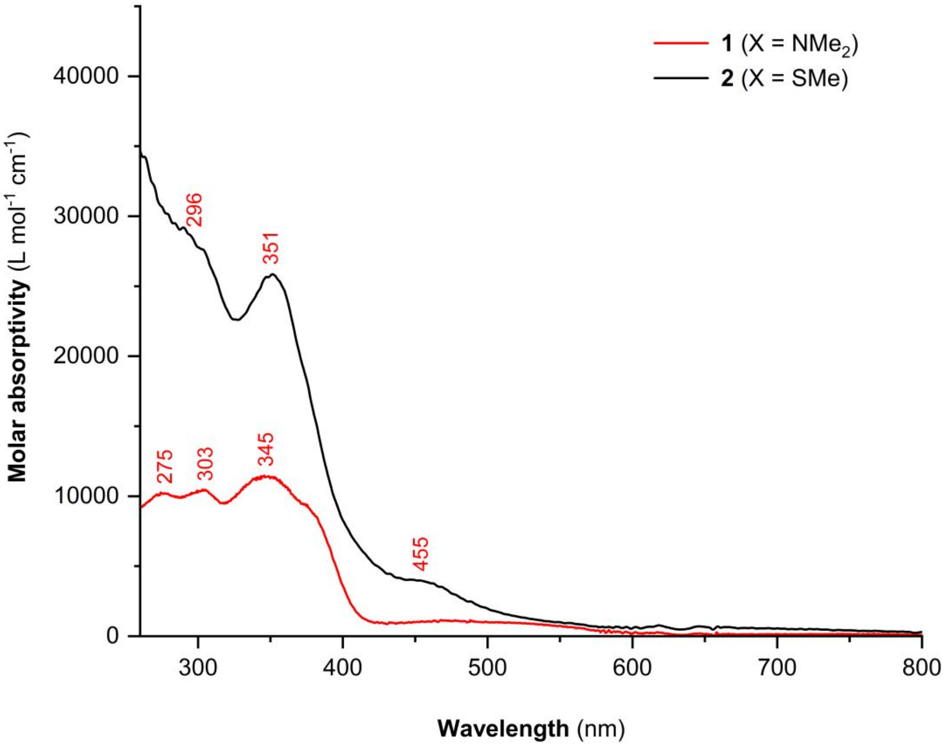

Figure S14. UV-vis spectra of Cr(**L1**) (**1**) and $[Cr(\mathbf{L2})_2]$ in thf.

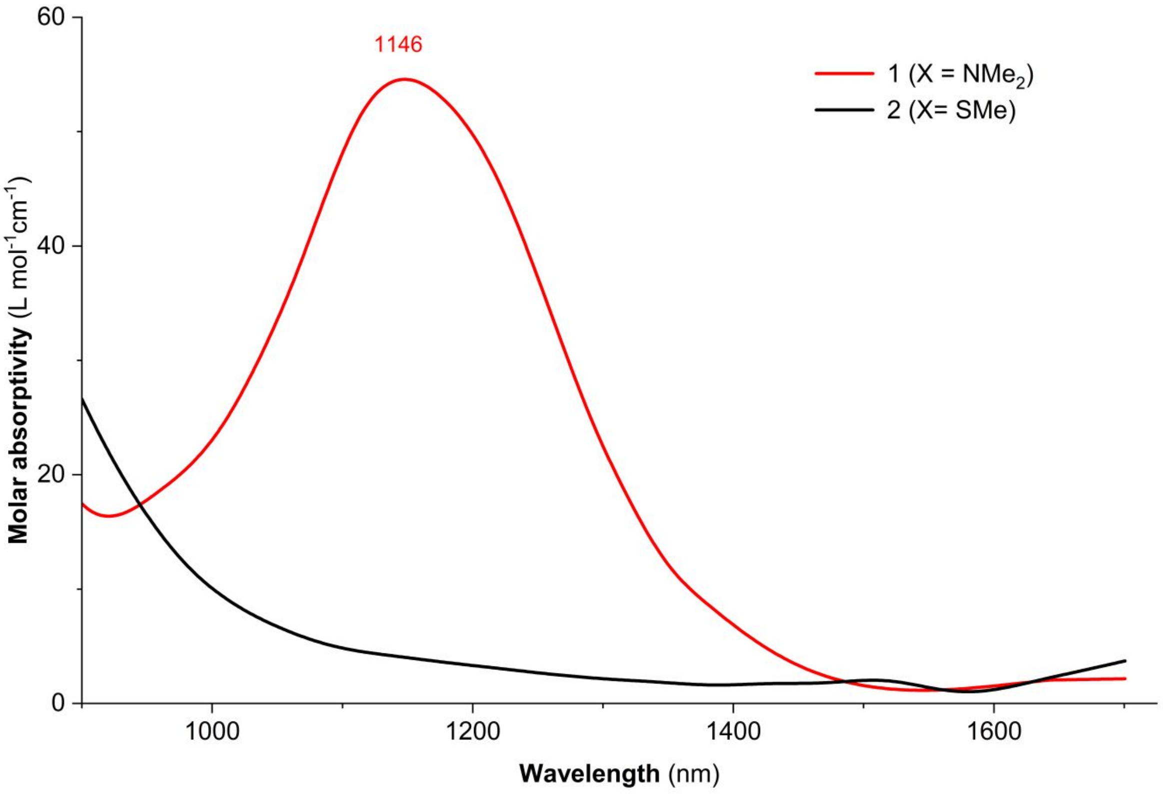

Figure S15. NIR spectra of Cr(**L1**) (**1**) and [Cr(**L2**)]$_2$ (**2**) in thf.

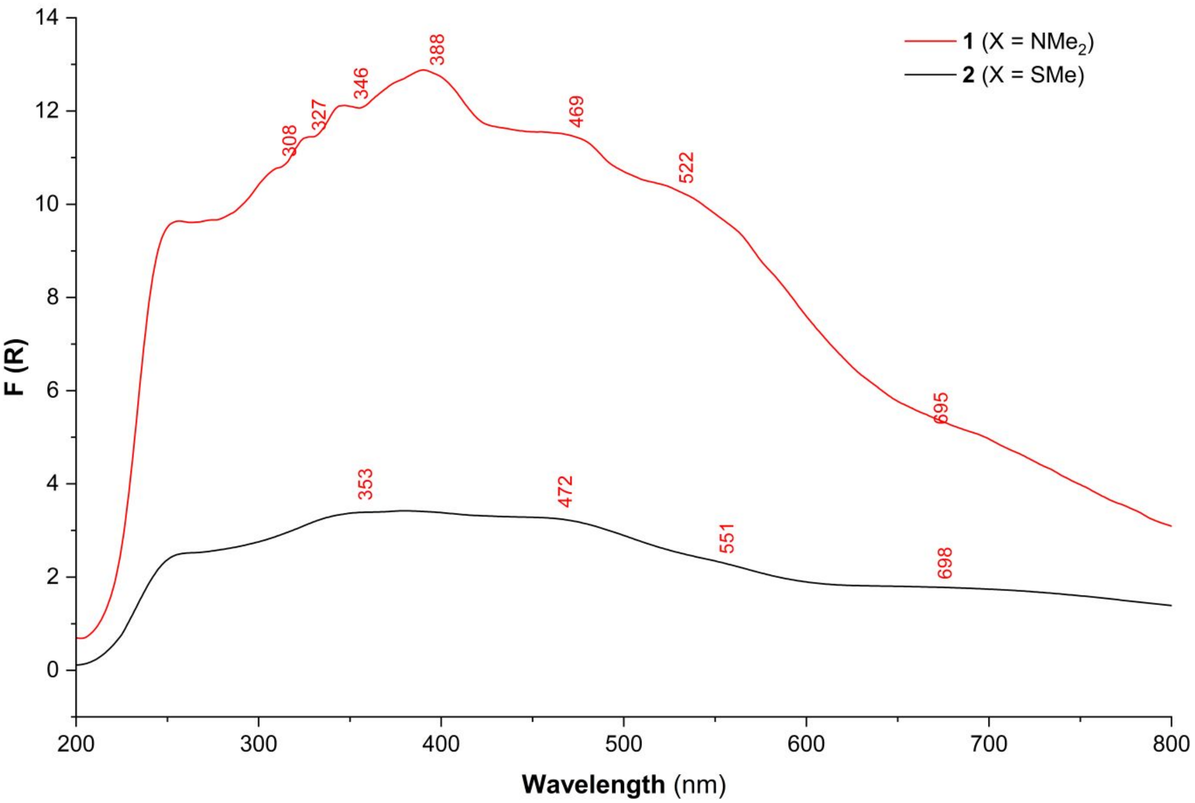

Figure S16. Solid-state UV-vis diffuse reflectance spectrum of Cr(**L1**) (**1**) and $[Cr(\mathbf{L2})]_2$ (**2**) plotted as Kubelka-Munk function F (R) vs. wavelength (nm). F (R) = $(1\text{-}R)^2/2R$ where R is diffuse reflectance.

8. ^{1}H NMR Spectra of 1 – 3

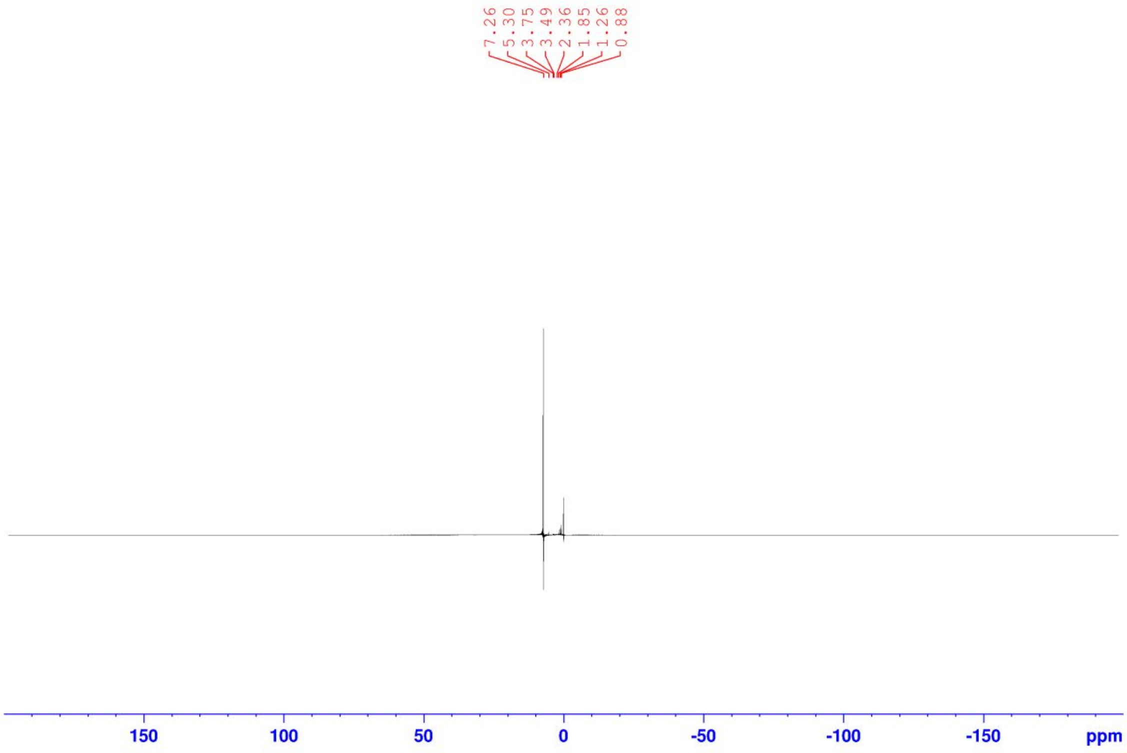

Figure S17. ^{1}H NMR spectrum of Cr(**L1**) (**1**) in $CDCl_3$. No obvious NMR resonances are observed due to the paramagnetism of the sample. The peaks observed are due to residual solvent and grease.

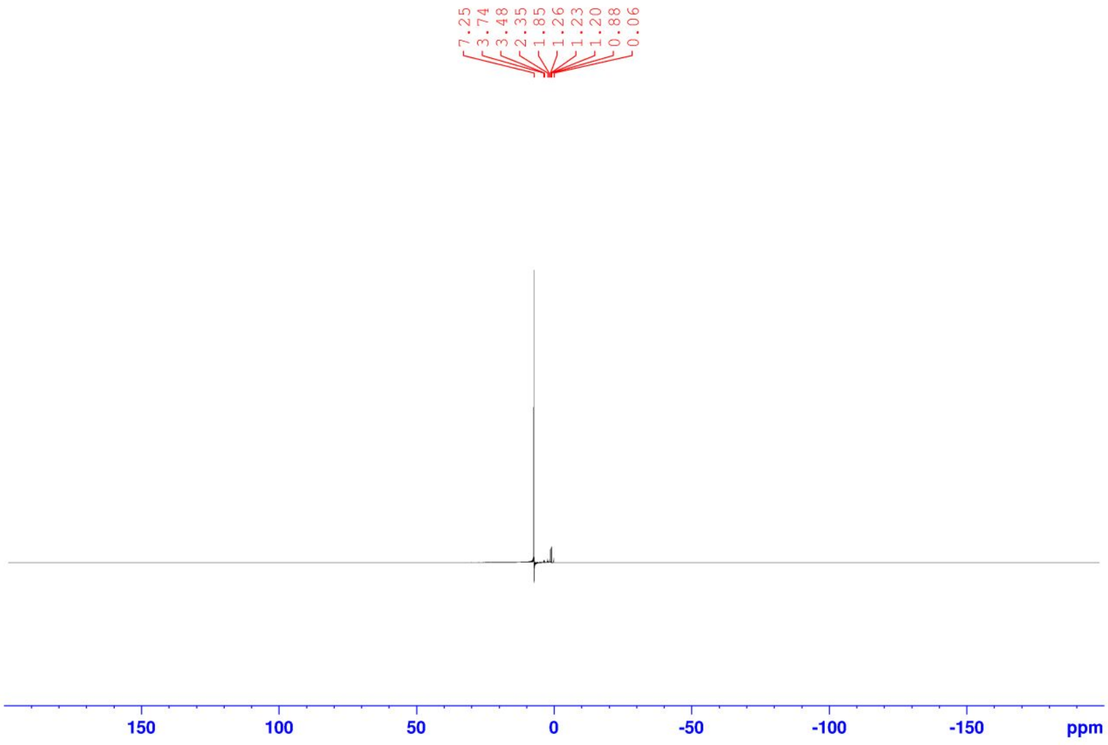

Figure S18. ^{1}H NMR spectrum of Cr(**L2**) (**2**) in $CDCl_3$. No obvious NMR resonances are observed due to the paramagnetism of the sample. The peaks observed are due to residual solvent and grease.

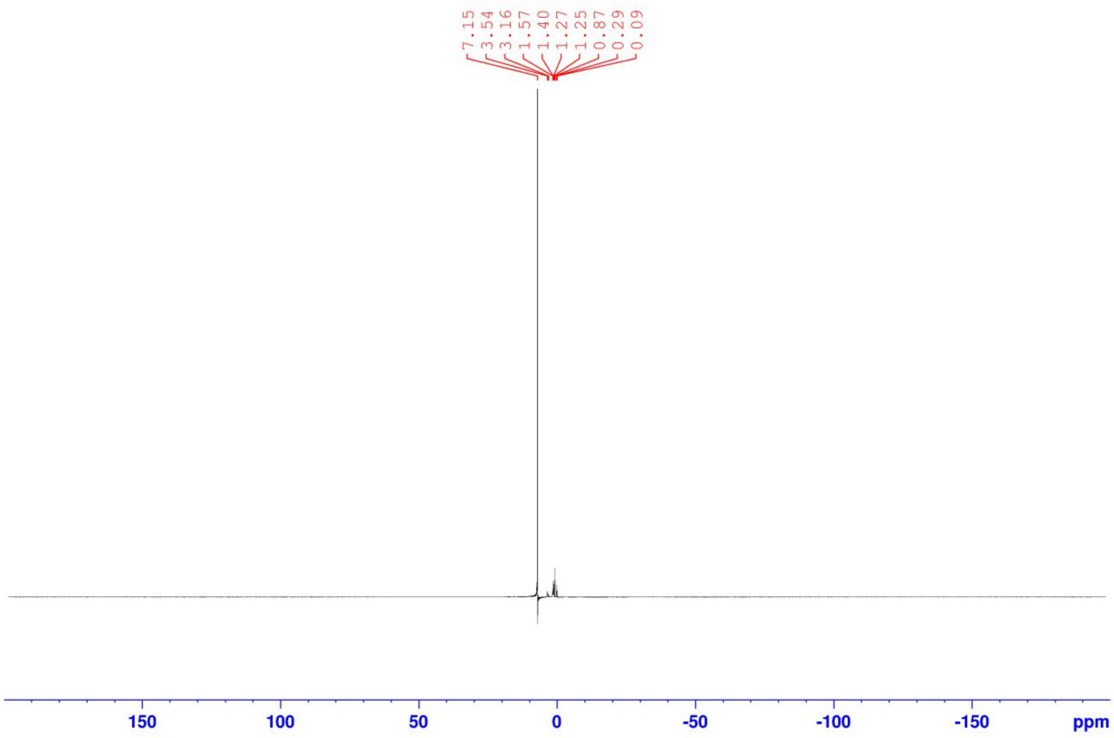

Figure S19. ^{1}H NMR spectrum of Cr(**L3**) (**3**) in C_6D_6. No obvious NMR resonances are observed due to the paramagnetism of the sample. The peaks observed are due to residual solvent and grease.
